# Characterization of the *Achromobacter xylosoxidans* Type VI Secretion System and Its Implication in Cystic Fibrosis

**DOI:** 10.3389/fcimb.2022.859181

**Published:** 2022-06-16

**Authors:** Mélanie Le Goff, Manon Vastel, Régine Lebrun, Pascal Mansuelle, Ava Diarra, Teddy Grandjean, Pauline Triponney, Geneviève Imbert, Philippe Gosset, Rodrigue Dessein, Fabien Garnier, Eric Durand

**Affiliations:** ^1^ Laboratoire d’Ingénierie des Systèmes Macromoléculaires (LISM), Institut de Microbiologie, Bioénergies et Biotechnologie (IM2B), Aix-Marseille Université - Centre National de la Recherche Scientifique (CNRS), Unité Mixte de Recherche (UMR) 7255, Marseille, France; ^2^ Université de Limoges, INSERM, Centre Hospitalier Universitaire (CHU) Limoges, Unité Mixte de Recherche (UMR) 1092, Limoges, France; ^3^ Plateforme Protéomique de l’Institut de Microbiologie de la Méditerranée, Marseille Protéomique, Aix Marseille Université, Centre National de la Recherche Scientifique (CNRS) FR 3479, Marseille, France; ^4^ Université de Lille, Centre National de la Recherche Scientifique (CNRS), INSERM, Centre Hospitalier Universitaire (CHU) Lille, Institut Pasteur de Lille, U1019-Unité Mixte de Recherche (UMR) 9017-CIIL-Centre d’Infection et d’Immunité de Lille, University of Lille, Lille, France; ^5^ Centre National de Référence de la Résistance aux Antibiotiques , Centre Hospitalier Universitaire de Besançon, Besançon, France; ^6^ Laboratoire Bactériologie, Hôpital Sainte Musse, Toulon, France; ^7^ Laboratoire d’Ingénierie des Systèmes Macromoléculaires (LISM), Institut de Microbiologie, Bioénergies et Biotechnologie (IM2B), Aix-Marseille Université - Unité Mixte de Recherche (UMR) 7255, INSERM, Marseille, France

**Keywords:** bacterial secretion system, T6SS, virulence factor, cystic fibrosis, bacterial competition, clinical microbiology

## Abstract

Bacteria of the genus *Achromobacter* are environmental germs, with an unknown reservoir. It can become opportunistic pathogens in immunocompromised patients, causing bacteremia, meningitis, pneumonia, or peritonitis. In recent years, *Achromobacter xylosoxidans* has emerged with increasing incidence in patients with cystic fibrosis (CF). Recent studies showed that *A. xylosoxidans* is involved in the degradation of the respiratory function of patients with CF. The respiratory ecosystem of patients with CF is colonized by bacterial species that constantly fight for space and access to nutrients. The type VI secretion system (T6SS) empowers this constant bacterial antagonism, and it is used as a virulence factor in several pathogenic bacteria. This study aimed to investigate the prevalence of the T6SS genes in *A. xylosoxidans* isolated in patients with CF. We also evaluated clinical and molecular characteristics of T6SS-positive *A. xylosoxidans* strains. We showed that *A. xylosoxidans* possesses a T6SS gene cluster and that some environmental and clinical isolates assemble a functional T6SS nanomachine*. A. xylosoxidans* T6SS is used to target competing bacteria, including other CF-specific pathogens. Finally, we demonstrated the importance of the T6SS in the internalization of *A. xylosoxidans* in lung epithelial cells and that the T6SS protein Hcp is detected in the sputum of patients with CF. Altogether, these results suggest for the first time a role of T6SS in CF-lung colonization by *A. xylosoxidans* and opens promising perspective to target this virulence determinant as innovative theranostic options for CF management.

## Introduction

Bacterial lung infections are the major cause of morbidity and mortality in patients with cystic fibrosis (CF) (O’Sullivan and Freedman, 2009). One of the most frequently involved bacterial pathogens is *Pseudomonas aeruginosa*, which infects over 37.5% of patients with CF (Crull et al., 2018; French Cystic Fibrosis Foundation’s Report, 2019; https://www.vaincrelamuco.org/). Other multi-resistant pathogens such as *Achromobacter xylosoxidans*, *Stenotrophomonas maltophilia*, or *Burkolderia cepacia* complex are also isolated. These emerging bacteria are still at a low prevalence, but they are increasing gradually over time (Mahenthiralingam, 2014; CysticFibrosisFundation, 2015; Parkins and Floto, 2015).

Although *Achromobacter* species are strict aerobic Gram-negative bacilli, ubiquitous, and widely distributed in aquatic environments and soil and also in the gut microbiota of healthy people, they are increasingly isolated in nosocomial settings (Swenson and Sadikot, 2015). Indeed, in immunocompromised patient, *Achromobacter* species can be involved in pneumoniae, urinary tract infections, digestive infections, meningitidis, and eyes infections (Swenson and Sadikot, 2015). They may also act as opportunistic pathogens in subjects with CF, where these microorganisms can survive over long periods in both airways and gut. In the last decade, *Achromobacter* spp. gained attention as important emerging pathogens that can cause severe acute and chronic infections in patients with CF (Hansen et al., 2010; Swenson and Sadikot, 2015), and *Achromobacter xylosoxidans* is the most frequently isolated species among patients with CF, followed by *Achromobacter ruhlandii*, *Achromobacter insuavis*, *Achromobacter insolitus*, *Achromobacter dolens*, *Achromobacter agrifaciens*, and *Achromobacter spanius* (Barrado et al., 2013; Spilker et al., 2013; Amoureux et al., 2016; Edwards et al., 2017; Gade et al., 2017; Papalia et al., 2020). More than half of patients with CF whose airways are colonized by *A. xylosoxidans* develop chronic infections, which have been associated with impaired respiratory function and lung inflammation (Hansen et al., 2010; Lambiase et al., 2011; Pereira et al., 2011; Firmida et al., 2016). The management of these infections and strains eradication is further complicated by the innate and acquired multidrug resistance of the strains (Traglia et al., 2012; Hu et al., 2014). Approximately 50 drug-resistance-associated genes have been predicted in the *A. xylosoxidans* type strain ATCC 27061 (Hu et al., 2015).

Although little is known about the clinical impact of *A. xylosoxidans* in CF, the presence of this pathogen is involved in the increase of lung inflammation and increased frequency of exacerbations, as well as more severe pulmonary infections (Hansen et al., 2010; Firmida et al., 2016; Marsac et al., 2021). On the other hand, the installation process of *A. xylosoxidans* strains is not fully clarified nor does their behavior within the respiratory microbiota of patients with CF, particularly with respect to other bacteria such as *S. aureus* and *P. aeruginosa*.

The virulence of *A. xylosoxidans* relies on its arsenal of virulence factors, whose only few have already been experimentally characterized (Menetrey et al., 2021). *A. xylosoxidans* expresses cell membrane-bound virulence factors such as proteins responsible for adhesion (Li et al., 2013), components promoting biofilm formation (Nielsen et al., 2016), proteins involved in the implantation in the mucus, proteins modulating the action of the immune system (Hutchison et al., 2000; Mantovani et al., 2012; Ridderberg et al., 2015), and the inhibitory colicin V, a protein cytotoxic to similar bacteria (Jakobsen et al., 2013). *A. xylosoxidans* is also equipped with various secretion systems (type I to IV) that mediate the release of molecules able to provide host cells invasion capability, but little is known about these exoproducts (Jakobsen et al., 2013; Ridderberg et al., 2015). For instance, genes encoding the type III secretion system (T3SS) are more common in CF isolates than in environmental strains (Li et al., 2013; Jeukens et al., 2017).

Bioinformatics analysis speculated the presence of type VI secretion system (T6SS) genes in *Achromobacter* spp. Twelve genes encoding for T6SS were identified in the genome of *A. xylosoxidans* strain NH44784-1996, an isolate from a patient with CF isolated in 1996 at the Copenhagen CF Centre (Jakobsen et al., 2013; Menetrey et al., 2021). Moreover, an *Achromobacter* spp. isolate produced exoproducts that interfered with the adhesion ability of a co-isolated *P. aeruginosa* strain and thus affected its ability to set up biofilm. The genome of this *Achromobacter* spp. isolate possesses a gene encoding a type VI secretion VgrG component (Sandri et al., 2021).

**Figure 1:**
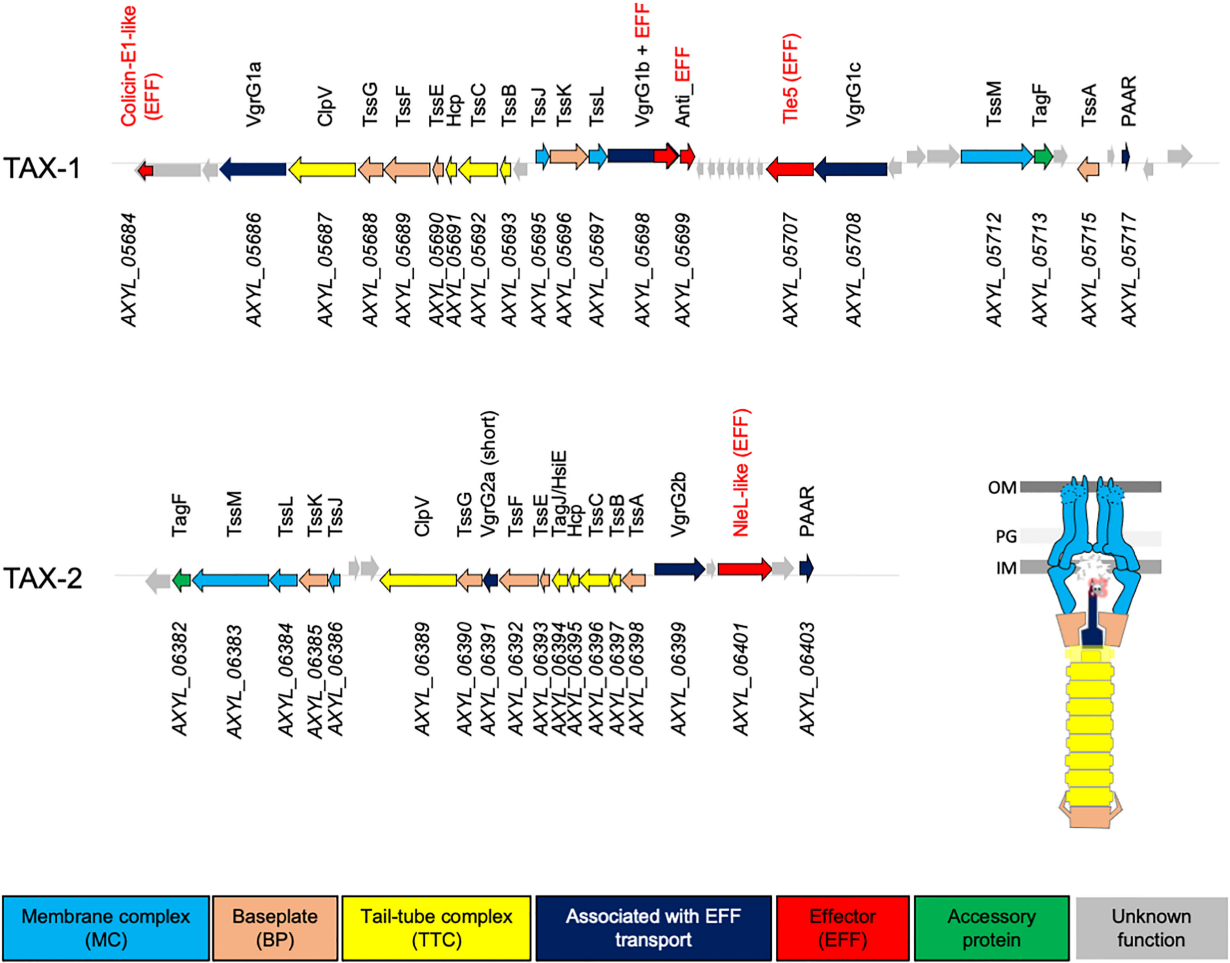
Genetic organization of the TAX-1 and TAX-2 operons in A8. Genes depicted as arrows are labeled with annotated numbers (below) and when applicable (homologous to known T6SS gene), the given name according Tss/Tag protein nomenclature (above). Arrows are color-coded based on predicted function: membrane complex (MC), baseplate (BP), and tail-tube complex (TTC), associated with EFF transport, effector (EFF), accessory protein, and unknown function are colored in light blue, orange, yellow, dark blue, red, green, and gray, respectively. The scheme represents the architectural model of the T6SS nanomachine.

The T6SS is a bacterial “nanomachine” that is present in 25% of Gram-negative bacteria, including in respiratory pathogenic bacteria, such as *P. aeruginosa*. It is instrumental for the colonization of their ecological niche and for promoting acute and chronic respiratory infections (Azam and Khan, 2019). These pathogens use T6SS to kill other bacteria, so they can take “control” of the respiratory microbiota, and to trigger their own internalization in pulmonary epithelial cells (Jiang et al., 2014; Sana et al., 2016; Allsopp et al., 2020). The T6SS bacterial nanomachine comprises 13–15 proteins, which combination results in a structure anchored in the bacterial envelope (**Figure 1**) (Zoued et al., 2014). Three architectural sub-complexes can be described. Four proteins (TssB, TssC, Hcp, and ClpV) regulate the function of a cytoplasmic contractile structure, the tail-tube complex (“TTC”). TTC assembly is coordinated by a complex of six proteins (TssK, TssE, TssF, TssG, VgrG, and TssA), the baseplate (“BP”), which acts also as a sorting platform for toxins. The BP complex is anchored to the bacterial membrane thanks to the interaction with a complex of four proteins (TssJ, TssL, TssM, and TagL), which is called the membrane complex (“MC”).

T6SS has been identified as a novel virulence factor for a number of clinically relevant pathogens. Although the *A. xylosoxidans* genome potentially encodes for a T6SS nanomachine, no study has directly investigated its utilization by the pathogen, especially in the CF context. The purpose of this study was to investigate the presence and the distribution of the T6SS genes in a clinical strains collection of *Achromobacter* and further analyze its clinical and molecular characteristics in representative *A. xylosoxidans* strains. In this study, we identified T6SS systems in all *A. xylosoxidans* clinical isolates. We demonstrated that one of these systems, TAX-1, is involved in the secretion of toxin and is used to kill other bacterial species, mostly competitive pathogens inhabiting the same niche. We demonstrated the implication of TAX-1 in the internalization of *A. xylosoxidans* in alveolar basal epithelial cells from Human adenocarcinoma. Finally, we proved the presence of a past T6SS activity in sputum collected from patients with CF colonized by *A. xylosoxidans.* The TAX-1 may therefore be considered as a key pathogenic factor promoting niche establishment of *A. xylosoxidans* in patients with CF.

## Results

### Identification of T6SS Genes in a Collection *A. xylosoxidans* Strains

A previous study has reported the presence of T6SS-encoding genes in the genome of a clinical strain of *A. xylosoxidans*, NH44784-1996, isolated from a patient with CF (Jakobsen et al., 2013). To determine whether this feature is conserved in the core genome of *A. xylosoxidans*, we used the KEGG database (https://www.genome.jp/kegg, T01341) and assayed the whole genome of the haloaromatic acid–degrading *A. xylosoxidans* A8 strain (GenBank GCA_000165835.1; Strnad et al., 2011) for the presence of T6SS operons. The resultant genome area was screened for genes and/or proteins that showed sequence homology with genes or proteins associated with T6SS. This targeted search resulted in the identification of a gene cluster of 18 open reading frames. Using this approach, we identified genes that are predicted to encode 12 of the 13 core T6SS proteins (Tss, Hcp, and VgrG) and two accessory structural or regulatory components that are present in some, but not all, T6SSs (Coulthurst, 2019). Interestingly, we detected two T6SS-encoding operons that we named **T**6SS ***A***. ***x*** *ylosoxidans* (TAX)-1 and TAX-2 (**Figure 1**). Both TAX encompass the complete sets of T6SS genes encoding for the MC, the BP, the TTC, and four effectors (EFF), which suggested their potential functional utilization by the *A. xylosoxidans* A8 strain.

To explore the clinical relevance of these T6SS, we set up a collection of 42 strains (37 A*. xylosoxidan*s, four *Achromobacter* spp., and one *A. insuavis*) from various sources (Limoges University Hospital, Toulon General Hospital, and the Reference National Center of the Antibiotics Resistance). This collection of clinical (CF sputum = 7; non-CF sputum = 7; blood = 7; other sites—tracheal aspiration, wound, bronchial aspirate liquid, catheter, aneurysm of aorta, ear, urine, and stools = 18) and environmental = 3 strains was screened for the presence of TAX-1 and TAX-2 operons using PCR primers designed on the *A. xylosoxidans* A8 genome (**Table S1**). We detected the presence of *tssC1*, *tssE1*, *tssM1*, and *hcp1* (from TAX-1) in all *A. xylosoxidan*s and *A. insuavis* isolates, but in only one *Achromabacter* spp. strain, independently of their origin. On the contrary, only the *A. xylosoxidans* A8 strain was positive for the presence of the TAX-2 genes (*tssC2*, *tssM2*, and *hcp2*) (**Table 1**). We then focus our study on three strains representative of *A. xylosoxidans*, A8, Ax4 (an expectoration CF strain) and Ax6 (a bloodstream strain).

**Table 1:** Genomic survey of T6SS distribution in *A. xylosoxidans* clinical isolates and clinical characteristics.

	Origin	Species	TAX-1 (positive PCR)	TAX-2 (positive PCR)
A8	DSMZ	*A. xylosoxidans*	*hcp1*, *tssC1*, *tssE1*, *tssM1*	*hcp2*, *tssC2*, *tssM2*
Ax2	Blood	*A. xylosoxidans*	*hcp1*, *tssC1*, *tssE1*, *tssM1*	*-*
Ax3	CF sputum	*A. xylosoxidans*	*hcp1*, *tssC1*, *tssE1*, *tssM1*	*-*
Ax4	CF sputum	*A. xylosoxidans*	*hcp1*, *tssE1*, *tssM1*	*-*
Ax5	CF sputum	*A. xylosoxidans*	*hcp1*, *tssC1*, *tssE1*, *tssM1*	*-*
Ax6	Blood	*A. xylosoxidans*	*hcp1*, *tssC1*, *tssE1*, *tssM1*	*-*
Ax7	CF sputum	*A. xylosoxidans*	*hcp1*, *tssC1*, *tssE1*, *tssM1*	*-*
Ax8	CF sputum	*A. insuavis*	*hcp1*, *tssC1*, *tssE1*, *tssM1*	*-*
Ax9	CF sputum	*A. xylosoxidans*	*hcp1*, *tssC1*, *tssE1*, *tssM1*	*-*
Ax11	Environmental	*A. xylosoxidans*	*hcp1*, *tssC1*, *tssE1*, *tssM1*	*-*
Ax12	Environmental	*A. xylosoxidans*	*hcp1*, *tssC1*, *tssE1*, *tssM1*	*-*
Ax13	Environmental	*A. xylosoxidans*	*hcp1*, *tssC1*, *tssE1*, *tssM1*	*-*
Ax14	ND^a^	*A. xylosoxidans*	*hcp1*, *tssC1*, *tssE1*, *tssM1*	*-*
Ax15	ND	*A. xylosoxidans*	*hcp1*, *tssC1*, *tssE1*, *tssM1*	*-*
Ax16	ND	*A. xylosoxidans*	*hcp1*, *tssC1*, *tssE1*, *tssM1*	*-*
Ax17	ND	*A. xylosoxidans*	*hcp1*, *tssC1*, *tssE1*, *tssM1*	*-*
Ax18	ND	*A. xylosoxidans*	*hcp1*, *tssC1*, *tssE1*, *tssM1*	*-*
Ax19	ND	*A. xylosoxidans*	*hcp1*, *tssC1*, *tssE1*, *tssM1*	*-*
Ax20	CF sputum	*Achromobacter spp.*	*-*	*-*
Ax21	Blood	*A. xylosoxidans*	*hcp1*, *tssC1*, *tssE1*, *tssM1*	*-*
Ax22	Non-CF sputum	*A. xylosoxidans*	*hcp1*, *tssC1*, *tssE1*, *tssM1*	*-*
Ax23	Blood	*A. xylosoxidans*	*hcp1*, *tssC1*, *tssE1*, *tssM1*	*-*
Ax24	Blood	*A. xylosoxidans*	*hcp1*, *tssC1*, *tssE1*, *tssM1*	*-*
Ax25	Non-CF sputum	*A. xylosoxidans*	*hcp1*, *tssC1*, *tssE1*, *tssM1*	*-*
Ax26	Non-CF sputum	*Achromobacter spp.*	*hcp1*, *tssC1*, *tssE1*, *tssM1*	*-*
Ax27	Urine	*A. xylosoxidans*	*hcp1*, *tssC1*, *tssE1*, *tssM1*	*-*
Ax28	Tracheal aspiration	*A. xylosoxidans*	*hcp1*, *tssC1*, *tssE1*, *tssM1*	*-*
Ax29	Non-CF sputum	*A. xylosoxidans*	*hcp1*, *tssC1*, *tssE1*, *tssM1*	*-*
Ax30	Non-CF sputum	*A. xylosoxidans*	*hcp1*, *tssC1*, *tssE1*, *tssM1*	*-*
Ax31	Wound	*A. xylosoxidans*	*hcp1*, *tssC1*, *tssE1*, *tssM1*	*-*
Ax32	ND	*A. xylosoxidans*	*hcp1*, *tssC1*, *tssE1*, *tssM1*	*-*
Ax33	Non-CF sputum	*A. xylosoxidans*	*hcp1*, *tssC1*, *tssE1*, *tssM1*	*-*
Ax34	Bronchial aspirate Liquid	*A. xylosoxidans*	*hcp1*, *tssC1*, *tssE1*, *tssM1*	*-*
Ax35	Catheter	*A. xylosoxidans*	*hcp1*, *tssC1*, *tssE1*, *tssM1*	*-*
Ax36	Blood	*Achromobacter spp.*	*-*	*-*
Ax37	Aneurysm of aorta	*A. xylosoxidans*	*hcp1*, *tssC1*, *tssE1*, *tssM1*	*-*
Ax38	Tracheal aspiration	*A. xylosoxidans*	*hcp1*, *tssC1*, *tssE1*, *tssM1*	*-*
Ax39	ND	*A. xylosoxidans*	*hcp1*, *tssC1*, *tssE1*, *tssM1*	*-*
Ax40	Ear	*A. xylosoxidans*	*hcp1*, *tssC1*, *tssE1*, *tssM1*	*-*
Ax41	Blood	*A. xylosoxidans*	*hcp1*, *tssC1*, *tssE1*, *tssM1*	*-*
Ax42	Non-CF sputum	*A. xylosoxidans*	*hcp1*, *tssC1*, *tssE1*, *tssM1*	*-*
Ax43	Urine	*A. xylosoxidans*	*hcp1*, *tssC1*, *tssE1*, *tssM1*	*-*
Ax44	Stools	*Achromobacter spp.*	*-*	*-*

^a^: Not dertermined.

### The Type VI Secretion System of *A. xylosoxidans* 1 (TAX-1) Is Active in Clinical Isolates

During T6SS functioning, the contraction of the TTC propels the Hcp-tube protein, together with the tip protein VgrG and effectors, both into the prey cells and outside in the extracellular space. These proteins are considered as a hallmark of a functional T6SS (Jurėnas and Journet, 2021). To correlate the presence of T6SS genes with the production of T6SS effectors, we analyzed the extracellular secretome of *A. xylosoxidans* strains using mass spectrometry–based proteomics. Only the A8 and the CF strains (Ax4), but not the bloodstream strain (Ax6), presented TAX-1 secreted products (Hcp_1) in their culture supernatant (**Table S2**). No TAX-2 exoproduct was detected in all tested strains (data not shown). In conclusion, TAX-1 appears to be active in the *A. xylosoxidans* reference strain A8 and in clinical isolates from CF patient.

### CF-Mimicking Conditions Trigger TAX-1 Activation in *A. xylosoxidans*

**Figure 2:**
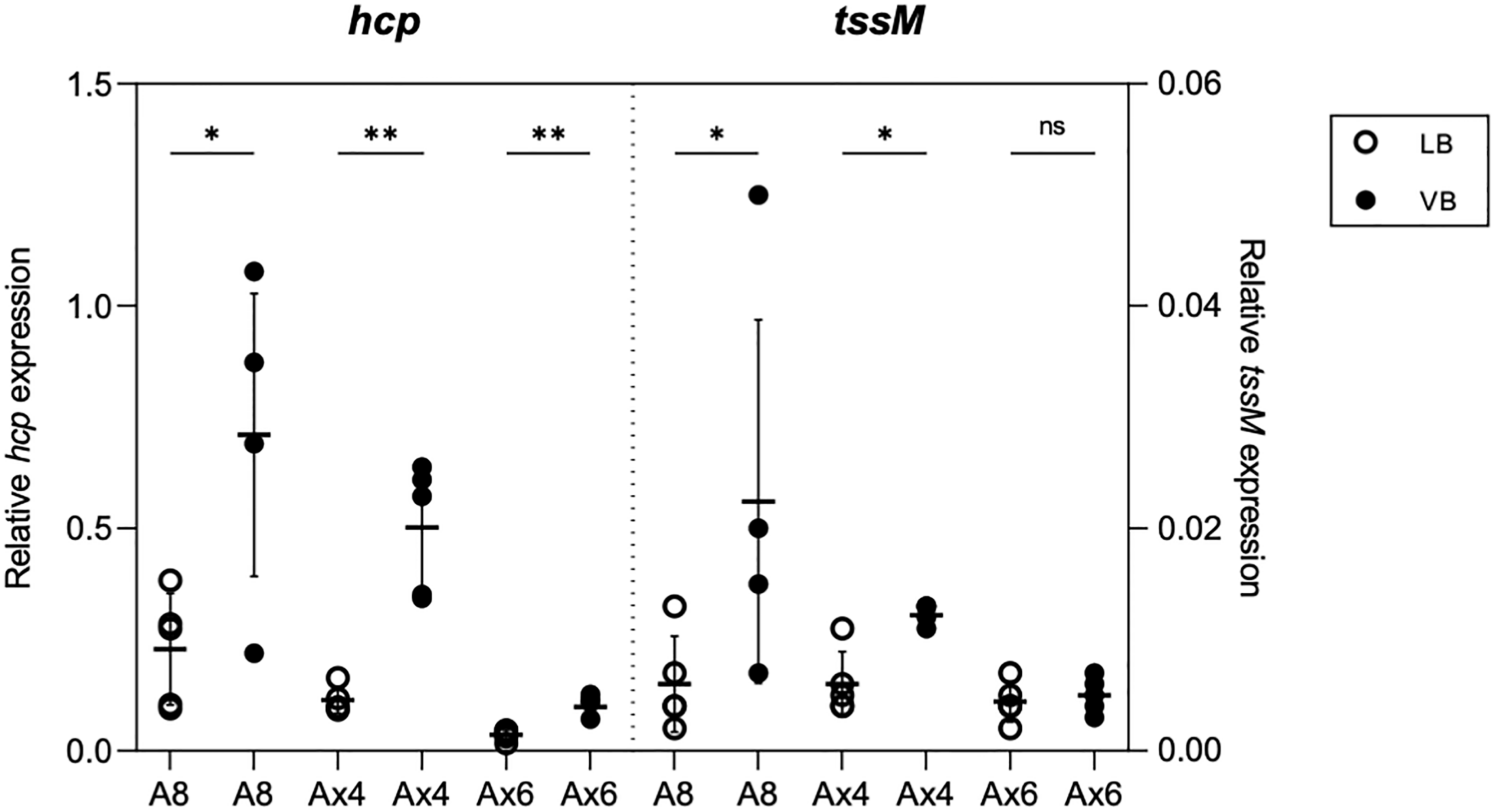
Effect of CF-mimicking medium VB on T6SS expression. qRT-PCR analysis of *hcp* and *tssM* of A8, Ax4, and Ax6 grown in LB (white circle) or VB (black circle). Data represented as circles from five biological replicates are normalized with the housekeeping gene *nrdA*. Horizontal bar and error bars represent the mean and standard deviation, respectively. *p < 0.05; **p < 0.01; ns, no significance.

Because of the production of a functional T6SS in the CF clinical isolate Ax4 but not in the bloodstream infection isolate Ax6, we investigated whether the specific conditions encountered by *A. xylosoxidans* in the CF lung could trigger T6SS activation. For this, Ax4 strain was cultured in both a rich medium (LB) and a CF-mimicking medium, i.e., the chemically defined Vogel–Bonner containing 19 mM amino acids as defined in synthetic CF sputum medium (VB, see Materials and Methods) (Palmer et al., 2007; Hood et al., 2010), and we performed a label-free relative quantitative mass spectrometry analysis on the secretome. The statistical T-test analysis (volcano plot) of the variations in the protein relative abundances revealed that extracellular products from the TAX-1 were specifically enriched in VB as compared to LB (**Figure S5**, red squares): Hcp, the three VgrG (1a, 1b, and 1c), PAAR, and a putative effector encompassing a ColE1-type of anti-bacterial toxin domain were overproduced (**Table 2**). Of note, the type III secretion system (T3SS) was activated in strains cultured on the CF mimicking medium (**Table 2** and **Figure S5**, green squares). Conversely, flagella-related products appeared to be less abundant in the CF-specific conditions (**Table 2** and **Figure S5**, blue squares). To discriminate between transcriptional or post-translational activation of the T6SS-secreted products observed in strains cultured on the CF mimicking medium, we performed a transcriptomic analysis of the TAX-1 genes. We quantified the transcripts of two genes, *hcp* and *tssM*, that are found in TTC and MC, respectively. These transcripts were normalized by comparison to the *nrdA* gene used as a house-keeping gene. For the three strains tested (A8, Ax4, and Ax6) and in both media, the *hcp* gene was more transcribed than the *tssM* gene (**Figure 2**). Interestingly, both TAX-1 *hcp* and *tssM* genes appeared to be induced by the CF mimicking medium in the A8 (*hcp*: 3.1-fold change VB vs. LB and *tssM*: 3.7) and Ax4 strains (*hcp*: 4.4 and *tssM*: 2), whereas this activation was not observed in the Ax6 blood stream isolate (*hcp*: 2.8 and *tssM*: 1.1) (**Figure 2**). These results were confirmed with a larger number of clinical isolates (**Figure S7**). Western blot analysis, using a specific antibody against TAX-1 Hcp (see Materials and Methods), was used to analyze whole-cell fraction (WCL) and concentrated culture supernatant (SN) fractions confirmed the induction of TAX-1 Hcp production and secretion by the CF-mimicking medium in the A8 and Ax4 strains (**Figure S1B** **)**. Conversely, no Hcp_1 was detected in the supernatant collected from the Ax6 strain, arguing that the transcript level detected was the background expression. In conclusion, proteomic and transcriptomic analyses validated the activation of the *A. xylosoxidans* TAX-1 in the CF-mimicking conditions.

**Table 2:** Secretomes from Ax4 grown in LB or VB were prepared in triplicate and subjected to mass spectrometry analysis.

	Putative Protein IDs	Unique peptides	Sequence coverage [%]	Fold change	Protein IDs
T6SS (TAX-1)	Colicin-E1–like effector	4	6.8	5.59	A0A0M7DM80
	VgrG1a	24	37	73.75	A0A0M7DL97
	Hcp1	13	75.4	6.92	A0A0D6HUB7;E3HG87
	VgrG1b + effector	4	2.8	12.04	E3HG94
	Effector domain	7	19.6	9.69	A0A0M7DKW2
	VgrG1c	32	47.6	50.74	A0A0M9I2Z4
	PAAR	3	8.8	5.31	A0A0M7DJN0
T3SS	CesT	4	29.6	8.53	A0A0M7IUG8
	SepL	11	35.2	11.15	A0A0M7KMF7
	EscI	6	42	2.85	A0A0M7KQZ8
Flagella	FliC	24	56.8	−3.00	A0A0M7L493
	FlgB	3	40.3	−2.83	A0A0M7M335;E3HSC4
	FlgC	9	89.9	−5.12	A0A0D6HEK3;E3HSC5
	FlgD	16	71.9	−3.95	A0A0M7KIR8;E3HSC6
	FlgE	14	45.5	−4.20	A0A0M7PS44;E3HSC7
	FlgF	12	64.8	−4.76	A0A0D6HCP9;E3HSC8
	FlgG	7	27.6	−5.31	A0A0M7M879;E3HSC9
	FlgK	18	37.5	−4.93	A0A0M7K095;E3HSD3
	FlgL	23	59.4	−4.67	A0A0D6HE69
	FliK	12	32.7	−5.23	A0A0M7F370;E3HSE7
	FliE	3	45.5	−4.65	A0A0D6HCW3;E3HSF3
	FliD	24	41	−2.87	A0A0M7D2V8
	FlaG	2	15.2	−6.12	A0A0M7NYY5

Protein included in TAX-1, T3SS, or flagella group are selected among significantly enriched in one growth condition.

### The TAX-1 Targets Anti-Bacterial Toxins to Competing Pathogens

**Figure 3:**
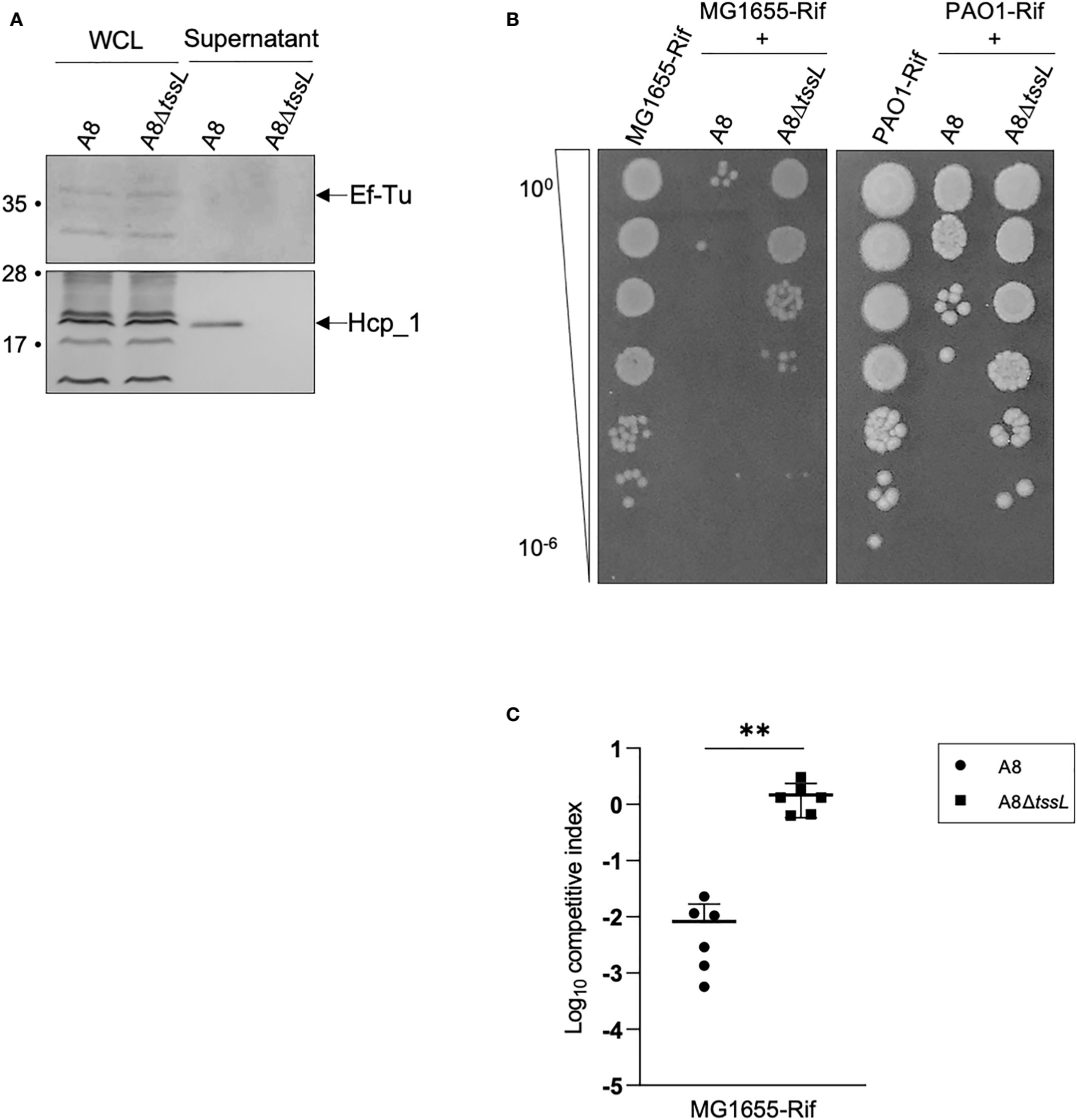
Deletion of *tssL* affects A8 Hcp secretion as well as its competition against *E. coli* and PAO1. **(A)** Immunoblot analysis of fractions containing 0.1 OD unit of whole-cell lysates (WCL) and 2 OD units of culture supernatants (SN) from *Achromobacter* A8 and A8Δ*tssL*. Ef-Tu was detected as cell lysis control. The position of Hcp and EF-Tu are indicated on the right. Molecular weight markers (in kilodaltons) are indicated on the left. **(B)** Picture of a representative survival of prey cells (MG1655-Rif or PAO1-Rif) after co-incubation with *Achromobacter* A8 or A8Δ*tssL*. Prey and attacker were mixed in a 1:10 ratio and incubated 6 h at 28°C on VB agar. Surviving preys are observed after spotting 10-fold serial dilutions of the mix on LB agar containing rifampicin. **(C)** MG1655-Rif were mixed with Achromobacter A8 or A8Δ*tssL* in a competition assay at 1:10 ratio. Both species were counted before and after 7 h of co-incubation to determine the competitive index (ratio of MG1655-Rif to Achromobacter output divided by the ratio of MG1655-Rif to Achromobacter input). Horizontal bars represent the mean and error bars represent the standard deviation. Statistical significance was determined by a Student’s unpaired *t*-test (**p = 0.0024).

To gain insights into the functionality of the TAX-1 in the A8 reference strain, we probed the presence of the Hcp tube protein by Western blot on both WCL and SN (see Materials and Methods). We demonstrated that the A8 strain produce and secrete Hcp in the supernatant (**Figure 3A**), thus confirming our previous proteomic results (**Table S2**). This observation indicates an active secretion rather than the consequence of cell leakage, because the EF-Tu signal is only detected in the WCL fraction (**Figure 3A**). To test whether this secretion is T6SS-dependent, we constructed a A8 mutant encompassing an in-frame deletion of the *tssL1* structural gene from TAX-1 (see Materials and Methods). The growth curves of strains A8 and A8Δ*tssL* were similar (**Figure S2**), indicating that *tssL* mutation did not significantly alter growth kinetics. The A8Δ*tssL* strain produces Hcp but is no longer able to secrete the protein in the supernatant (**Figure 3A**). Thus, we showed for the first time that the A8 strain uses the TAX-1 to actively secrete Hcp.

Using bioinformatic analysis, we predicted that the TAX-1 gene *AXYL_05707* could encode for PLD-like antibacterial effector that degrades phosphatidylethanolamine (PE) (**Figure S2**) (Russell et al., 2013). Indeed, a Pfam search showed that the AXYL_05707 effector possesses a domain duplication, which is apparent by the presence of two motifs containing well-conserved histidine, lysine, and/or asparagine residues. These residues may contribute to the active site (HKD1 and HKD2 domains) (**Figure S3**). Moreover, structural prediction using the HHpred for protein remote homology detection indicated that AXYL_05707 presented a degree of similarity to *Homo sapiens* phospholipase D1 (PDB 6U8Z, Bowling et al., 2020), sharing 31% identity (E-value: 1.8e-81). Interestingly, sequence alignment of AXYL_05707 with *P. aeruginosa* Tle5 and Tle5b (Jiang et al., 2014) showed conservation of catalytic residues, with AXYL_05707 looking closer to Tle5 than Tle5b (**Figure S3**). *P. aeruginosa* encodes the two phospholipases Tle5 and Tle5b that were shown to be delivered into prey cells in a H2_T6SS-dependent manner (Russell et al., 2013; Jiang et al., 2014; Boulant et al., 2018). This prediction prompted us to set up a competition assay between the A8 strain and *E. coli* MG1655 (see Materials and Methods). Interestingly, the A8 strain is able to kill *E. coli* at a 1:10 prey:attacker ratio, whereas the A8Δ*tssL* strain is still able to outcompete *E. coli* but far less efficiently (**Figures 3B, C** **,** **S6**). We thus concluded that the TAX-1 is responsible for the anti-bacterial activity of the *A. xylosoxidans* A8 strain.

In the lung, *A. xylosoxidans* encounters *P. aeruginosa*, another important pathogen associated with the worsening of the health conditions of patients with CF (Lambiase et al., 2011; Tetart et al., 2019). *P. aeruginosa* is able to outcompete other bacteria using the T6SS nanomachine (Chen et al., 2015; Sana et al., 2016). We tested whether *A. xylosoxidans* is able to kill *P. aeruginosa*. A bacterial competition assay was set up between the A8 strain and the *P. aeruginosa* PAO1 strain (see Materials and Methods). The A8 strain, but not the A8Δ*tssL* derivative, was able to kill PAO1 in a T6SS-dependent manner (**Figures 3B**, **S6**). We concluded that *A. xylosoxidans* uses the TAX-1 to fight other pathogens such as *P. aeruginosa* during the establishment of its niche in the CF lung.

### The TAX-1 Triggers Internalization of *A. xylosoxidans* in Human Cells

**Figure 4:**
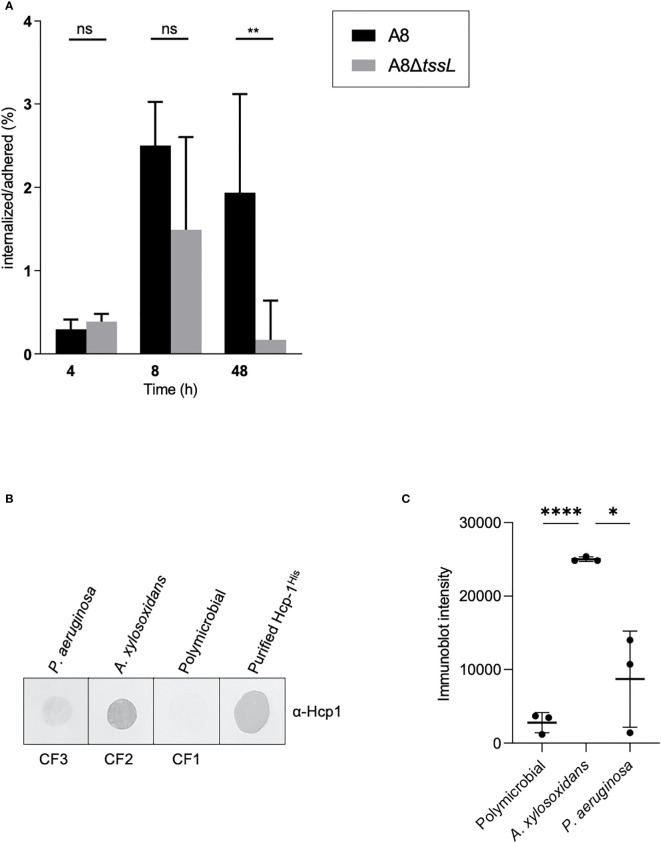
*Achromobacter* TAX-1 is active during (cystic fibrosis) infections. **(A)** Percentage of internalized on adhered bacteria of wild-type (WT) A8 and mutant A8Δ*tssL (*Δ*TSSL)* strain on A549 cells after 4, 8, and 48 h of infection at MOI 1:1. Values were expressed as median andinterquartile range. Comparison groups were analyzed with the nonparametric Mann–Whitney test. ** p < 0.01; ns, no significance. **(B)** Immunoblot of sputum from a patient with CF infected with either *A xylosoxidans* (CF2), *P. aeruginosa* (CF3), or with a commensal flora (CF1) analyzed with Hcp-1 antiserum. Purified Hcp-1^His^ protein served as positive control. **(C)** Immunoblot of sputum from a patient CF infected with either *A xylosoxidans* and *P. aeruginosa* or with a commensal flora were analyzed with Hcp-1 antiserum and quantified using ImageJ software. Horizontal bars represent the mean and error bars represent the standard deviation of n = 3 different sputums. Statistical significance was determined by a Student’s unpaired *t*-test. (****p < 0.0001; *p = 0.0124).

We have shown that the A8 TAX-1 encodes putative Tle5/Tle5b homologs. *P. aeruginosa* Tle5b is a trans-kingdom effector, targeting both prokaryotic and eukaryotic cells (Jiang et al., 2014). Moreover, in *P. aeruginosa*, the H2-T6SS Tle5b toxin triggers the internalization of the pathogen in lung epithelial cells (Sana et al., 2015; Wettstadt et al., 2019). This observation prompted us to investigate the function of the TAX-1 relative to the interaction of the pathogen with host cells. We infected human adenocarcinomic alveolar epithelial cells A549 with either A8 or A8Δ*tssL* strains and quantified the intracellular invasion. We estimated the percentage of internalized over adherent bacteria at 4, 8, and 48 h of infection. Raw calculation of internalized bacteria was approximately 10^3^, 10^4^, and 10^5^ UFC/ml, respectively, at 4, 8, and 48 h of infection. At 4 and 8 h of infection, there was no significant difference of internalization between wild-type A8 and mutant A8Δ*tssL*. However, we could observe a tendency of a lesser ratio of internalized over adherent bacteria in A8Δ*tssL-*infected cells at 8 h of infection. The percentage of internalized on adherent bacteria became significantly different between wild-type A8 and mutant A8Δ*tssL* at 48 h of infection of (*P* = 0.009) (**Figure 4A**). However, neither LDH release nor significant percentage of dead cells counted with trypan blue was observed in all conditions (**Figure S4B**), indicating the absence of cytotoxicity induced by the pathogen. These results suggest that the *A. xylosoxidans* TAX-1 participates in the internalization of the pathogen into human alveolar cells. In our model, T6SS-deficient *A. xylosoxidans* strains seem to be less internalized by lung epithelial cells than wild-type strains. However, the results are significant with a low amount of bacteria per cells and extended duration of infection. Of note, studied strains were deprived from T3 secretion system (T3SS), thus less cytotoxic.

### Detection of TAX-1 Activity in the Sputum of patients with CF

To address whether secretion of TAX-1 Hcp occurs naturally in *A. xylosoxidans*, we used Hcp_1-specific antibodies to directly examine Hcp secretion in the sputum of a patient with CF (**Figure 4B**). Three sputum samples were collected from three patients with CF: CF1, infected with *P. aeruginosa*; CF2, infected with *A. xylosoxidans*; and CF3, with a commensal flora. A dot blot using the anti-Hcp antibody was performed to evaluate the Hcp content in the pulmonary secretions. We have shown that Hcp was readily detected in sputum from a patient infected with *A. xylosoxidans* (CF2); however, the protein was not detected in sputum from a polymicrobial-infected patient (CF3) or a patient infected with *P. aeruginosa* (CF1). A quantification of the presence of Hcp in samples derived from multiple patients (three polymicrobial, three colonized by *A. xylosoxidans*, and three colonized by *P. aeruginosa*) confirmed our results (**Figure 4C**) Of note, our *A. xylosoxidans* Hcp antibody did not demonstrate any cross-reactivity with *P aeruginosa*-derived supernatant (**Figure S1C**). Thus, TAX-1 Hcp is actively secreted within the lungs of patients with CF who have had long-term infection, which supports a role for TAX-1 in chronic *A. xylosoxidans* infections.

## Discussion

*A. xylosoxidans* is increasingly becoming a threat to patients with CF. Indeed, this opportunistic pathogen is well equipped with several virulence factors that collectively promote the colonization of the host. In this work, we demonstrated that the genome of *A. xylosoxidans* A8 possesses two T6SS-encoding gene clusters that we named TAX-1 and TAX-2. However, we have shown that only TAX-1 is active in *A. xylosoxidans* A8 reference strain and in CF clinical isolates. We further demonstrated that CF-mimicking conditions trigger the activation of TAX-1, which resulted in anti-bacterial antagonism and internalization into human lung epithelial cells. Our study also indicates that the T6SS hallmark, the protein Hcp, is present in pulmonary secretions of patients with CF.

A new T6SS player in the “respiratory game”: The utilization of T6SS is widespread in respiratory pathogen. For instance, one of the first reports describing T6SS activity in *P. aeruginosa* demonstrated the presence of Hcp in patient’s sputum, as well as Hcp-specific antibody in their sera (Mougous et al., 2006). Members of the *Burkholderia cepacia* complex (Bcc) that cause devastating infections in patients with CF harbor a T6SS that is important for the infection of macrophages and influences the host immune response (Aubert et al., 2015; Aubert et al., 2016). Other respiratory pathogens, not implicated in CF, utilize the T6SS weapon. This is the case for *Acinetobacter baumannii* (Weber et al., 2017) and *Klebsiella pneumoniae* (Hsieh et al., 2019). Our findings that *A. xylosoxidans* TAX-1 is active in the lung of CF-patients provide another example of respiratory pathogens that utilize this versatile nanomachine. It also raises the hypothesis that, alongside other pathogens *A. xylosoxidans* uses its TAX-1 as a *bona fide* virulence factor.

One can envisage that an intense competition exists in the pulmonary tract between pathogens and also toward the resident microbiota, fighting for space and nutrient. These conflicts shape the composition of the healthy and pathogenic respiratory microbiota (Chassaing and Cascales, 2018) and most probably have a direct impact on the progression of the disease’s state. For instance, epidemic *Burkholderia cenocepacia* isolated from patients with CF can outcompete their co-isolated *P. aeruginosa* strains (Perault et al., 2020), which, in turn, makes the human host in danger when facing Bcc superinfection. Our observation that *A. xylosoxidans* can outcompete *P. aeruginosa* PAO1 in laboratory settings where the T6SS of the prey is active strongly encourages to investigate the clinical relevance in terms of consequences for the patient’s prognostic. Although *P. aeruginosa* appears to colonize various sites in the CF airways (Aanaes et al., 2011), whereas *A. xylosoxidans* seems to be restricted at the base of the lung (Garg et al., 2017), they both might endeavor at some stage the same journey through the respiratory tract and compete together, at least temporarily.

The dense mucus produced in the lung of patients with CF creates a micro-environment favorable to the development of a specific microbial community encompassing several pathogens (Burns et al., 1998; Lyczak et al., 2002; Ciofu et al., 2013). In this context, an intense battle orchestrates the interplay between the various species that compete for space and nutrient availability (Armbruster et al., 2019). Indeed, antagonistic interactions have been reported between *P. aeruginosa* and *S. aureus* (Hotterbeekx et al., 2017), or *Burkholderia cepacia* (Perault et al., 2020). As these interactions have major implications in the progression of polymicrobial pathologies, they are instrumental to understand (Limoli and Hoffman, 2019). Our study demonstrates that *A. xylosoxidans* utilizes the TAX-1 as an offensive weapon, targeting Gram-negative bacteria independently of their reciprocal attack by a T6SS. Notably, *A. xylosoxidans* is able to outcompete *P. aeruginosa*, another respiratory opportunistic pathogen. This observation is surprising, because bioinformatic inspections of the TAX-1 predict that it encodes several anti-bacterial toxins, of which a *P. aeruginosa*-Tle5/Tle5b homolog. Given that *P. aeruginosa* possesses an immunity protein to such kind of toxin, we were expecting that it would have been protected from killing by the Tle5-like effector. On the contrary, one can envision that *A. xylosoxidans* uses a different anti-bacterial toxin or a cocktail to target *P. aeruginosa*. The proteomic analysis of *A. xylosoxidans* supernatant revealed the presence of a multi-modular effector with a colicin ColE1-type of domain (Cramer et al., 1990). We will need to address the specific contribution of this TAX-1 effector in the killing of *P. aeruginosa*. Interestingly, *A. xylosoxidans* was previously reported to inhibit the growth and pigmentation of *P. aeruginosa*, whereas biofilm formation was drastically reduced (Menetrey et al., 2020), albeit without identifying the molecular mechanism. Our finding could point to the implication of TAX-1 in this fierce competition among CF-colonizing pathogens.

Here, we demonstrated that CF-mimicking conditions appear to upregulate TAX-1, together with T3SS. On the contrary, the flagella proteins are downregulated in the same conditions. In *P. aeruginosa*, the second messenger cyclic di-GMP controls the switch between acute and chronic mode of infection, mediated by the PA1611-RetS-GacS sensors (Moscoso et al., 2011; Kong et al., 2013; Chambonnier et al., 2016). This switch notably implies the downregulation of T3SS and flagella motility, required only during the acute infections, and the upregulation of T6SS and biofilm, which are important for the chronic mode of infection. *A. xylosoxidans* chronic infection in patients with CF is associated with a decline in their lung functions (Rønne Hansen et al., 2006). However, in various underlying illnesses, such as malignancies, cardiac disease or immunosuppressed patients, *A. xylosoxidans* can also cause acute infections, notably fatal bacteremia (Duggan et al., 1996). Moreover, it can occur even in apparently healthy individuals (Lee et al., 2016). Although we do not know yet the implication of the TAX-1 in acute versus chronic infections, its coregulation with the T3SS is meaningful. Indeed, the *A. xylosoxidans* T3SS transport the toxin AxoU that induces cytotoxicity in macrophages, which suggests a pathogenic or inflammatory role of T3SS in the CF lungs (Pickrum et al., 2020). Interestingly, internalization is required for *A. xylosoxidans* to induce cell death mediated by the T3SS. One can envisage that a “molecular collaboration” exists between T3SS and T6SS during this specific stage of the infection. Indeed, as reported for *P. aeruginosa* (Sana et al., 2015), TAX-1 could mediate the internalization of *A. xylosoxidans* into human cells where the activation of T3SS triggers the release of cytotoxic effectors. In support of this hypothesis, our bioinformatic study suggested that the TAX-1 could transport a trans-kingdom effector, AXYL_05707, similar to *P. aeruginosa* Tle5 or Tle5b that are involved in internalization. In *P. aeruginosa*, the production of Tle5 and ExoU is correlated with a high risk of exacerbations in non-CF patients (Boulant et al., 2018; Luo et al., 2019) and with acute pulmonary infection, septicemia and, in a lesser extent, UTIs (Boulant et al., 2018). Our findings, combined with the abovementioned recent observations, support the hypothesis that TAX-1, together with T3SS nanomachines, could be instrumental for *A. xylosoxidans* to develop acute infection in human. Further works are needed to prove the molecular connection between the two secretion systems, if any, and to demonstrate their production during infection settings. The more we will explore the fine molecular mechanism of bacterial nanomachines, the more we will discover interesting connection between them. Other examples of nanomachine cross-talk have been previously described. The H3-T6SS in *P. aeruginosa* seems to be crucial for the T3SS, pyocyanin production, biofilm formation, and *in vivo* pathogenicity (Li et al., 2020). A functional relationship between T6SS and flagella in *P. fluorescens* has been reported (Bouteiller et al., 2020) and, similarly, *Caulobacter crescentus* coordinates its unique cell cycle mechanism with the assembly of a single polar flagellum (Siwach et al., 2021).

Our finding that the specific micro-environment encounters by *A. xylosoxidans* in the lung of patients with CF could trigger the activation of TAX-1 questions the underlying molecular mechanism. Indeed, both the production and secretion of Hcp is more abundant in the CF-mimicking medium. Similarly, *hcp* and *tssM* transcripts are upregulated in the same conditions, demonstrating that the regulation is at the transcriptional level. In *P. aeruginosa*, human respiratory mucus activates the transcription of several virulence genes, such as catalases, peroxide-detoxifying enzymes, and T6SS (Cattoir et al., 2013), suggesting a potential role of T6SS during the infectious process. *A. xylosoxidans* shares common traits with other human pathogens in its capacity to respond and adapt to a human host, optimizing its long-term survival potential. *P. aeruginosa* has developed an intricate regulation network to adapt and to balance metabolism, virulence factors, and other cellular processes in response to its immediate environment (Pusic et al., 2021). Future works are needed to determine whether such adaptation occurs also in *A. xylosoxidans*, as suggested by our work, and to determine the players and the network they participate in. The detection of Hcp from *A. xylosoxidans* (present study) and *P. aeruginosa* (Mougous et al., 2006) in the sputum from patients with CF highlights the importance of this nanomachine in the CF context and paves the way for the development of new diagnostic tool and potential biomarker to identify CF pathogens. Surprisingly, we found that *A. xylosoxidans* blood strains possess T6SS-encoding genes but do not express a functional TAX-1 in any conditions tested. This finding raises questions about the mechanism involved in the silencing of the TAX-1 genes, which must be strong enough to guarantee their tight repression in changing environments. Previous works have evidenced such powerful regulatory mechanisms. In *P. aeruginosa*, T6SS-abrogating mutations are found in some clinical isolates, the repair of which reactivates T6SS (Perault et al., 2020). In the *A. baumannii* clinical isolate AbCAN2, a point mutation in the tail-tube component VgrG blocks the assembly of the T6SS (Lopez et al., 2020). The sequencing of the T6SS-gene cluster of blood isolates should bring molecular explanation to the reason why T6SS is inactive.

## Material and Methods

### Strains Collection

Our collection was constructed with 42 clinical and environmental strains from the following:

*Limoges university Hospital, n = 8 clinical strains, including six (five *A. xylosoxidans* and one *A. insuavis*) from the sputum of patients with CF and two *A. xylosoxidans* from blood culture of non-cystic fibrosis patients;
*Toulon general Hospital, n = 3 A*. xylosoxidans* environmental strains;
*The Reference National Center of the antibiotics resistance, n = 31 (27 A*. xylosoxidans* and four *Achromobacter* spp.) clinical strains, only one *A. xylosoxidans* came from patient with CF.

### Sputum Samples Collection and Processing

Nine sputum samples of CF patients were used in this study: three control without pathogenic bacteria, only a commensal flora , and six strains from pathogenic bacteria, three with A. xylosoxidans and three with *P. aeruginosa*.

### DNA Extraction From Clinical Isolates

For each strain, DNA was extracted using the SaMag-12^®^ system (Sacace Biotechnologies, Como, Italy) according to the manufacturer’s recommendations.

### Screening PCR

Screening PCR was carried out using primers presented in the **Table S1**, together with One Taq^®^ Quick-load (New England Biolabs, Évry-Courcouronnes, France) in a final volume of 12.5 µl with 0.5 µl of each primer at 10 µM, 6.25µl of One Taq buffer, and 0.5 µl of DNA extract. Amplification took place in the following conditions: 4 min at 95°C for initial denaturation, 30 cycles of 30 s at 95°C for denaturation, 30 s at 50°C for annealing, 30 s at 68°C for extension, and a final 7-min extension step at 68°C. PCR products were analyzed by electrophoresis in a 2% agarose gel in 0.5X TBE-containing Midori Green Advance DNA stain^®^ (Nippon Europe Genetics GmbH, Düren, German). A UVP gel documentation system was used for DNA band visualization.

### Quantification of Transcripts

Total RNA was extracted from sorted subpopulations of bacteria with the NucleoSpin^®^ kit (Machery Nagel, Hoerdt, France) following the manufacturer’s instructions. RNA concentration, quality, and integrity were evaluated with the Agilent RNA 6000 Pico^®^ kit on a Bioanalyzer. cDNA fragments were synthesized from 100 pg of total RNA using the PrimeScript RT^®^ reagent and perfect real time^®^ kit (Takara, Saint-Germain en Laye, France) with appropriate oligonucleotides (**Table S1**), following the manufacturer’s instructions. Assays were performed with a CFX96^®^ Touch detection system (Bio-Rad) using the following PCR cycling conditions: 3 min at 95°C and 40 cycles at 95°C for 15 s and 59°C for 1 min. Assays were performed in triplicate. The quantification of *hcp1* and *tssM* genes was normalized to the quantification of the housekeeping gene *nrdA*.

### Bacterial Strains and Growth Conditions

*E. coli* DH5α and BL21(DE3) were used for cloning procedures and protein production of Hcp-1, respectively. *E. coli* CC118λPir was used for cloning procedures and triparental mating with 1,047 pRK2013 helper strains to generate *tssL* mutant. *Achromobacter* spp. A8 (German collection of microorganisms and cell cultures GmbH, DSMZ, DSM-26587) was the reference strain used in this study, as well as the parental strain for *tssL* mutant. Rifampicin-resistant derivatives of PAO1 and MG1655 (laboratory collection) were isolated by plating wild-type strains on LB agar containing increasing concentration of rifampicin up to a final concentration 100 µg ml^−1^. PAO1-Rif and MG1655-Rif were used in interbacterial competition assay. All strains were maintained at 37°C on Luria Bertani (LB) agar. Liquid cultures were routinely grown at 37°C or 28°C in LB broth or VB (Vogel–Bonner minimal medium* VBC containing 0.16% glucose and 19 mM amino acids as defined in CF-mimicking medium; Vogel and Bonner, 1956; Palmer et al., 2007) with orbital shaking at 180 rpm. When required, rifampicin was used at a concentration of 100 µg ml^−1^, Kanamycin at 25 µg ml^−1^, and Streptomycin (Sm) at 50 µg ml^−1^ for *E. coli* and 2000 µg ml^−1^ for *Achromobacter* spp. A8. Gene expression from pRSF vector was induced with isopropyl-β-D-1-thio-galactopyranoside (IPTG). *VB: 10.4 mM citric acid, 16.7 mM NaNH_4_HPO_4_,4H_2_O, 57.4 mM K_2_HPO_4_, 0.8 mM MgSO_4_, 0.16% glucose, 0.827 mM L-aspartate, 1,072 mM L-threonine, 1.446 mM L-serine, 0.16 mM L-glutamate, 1.117 mM L-valine, 0.633 mM L-methionine, 0.013 mM L-tryptophan, 0.306 mM L-arginine-HCl, 1.12 mM L-isoleucine, 1.609 mM L-leucine, 0.802 mM L-tyrosine, 0.53 mM L-phenylalanine, 0.676 mM L-ornithine, 2.128 mM L-lysine, and 0.519 mM L-histidine-HCl.

### Mutagenesis—Construction of the pKNG101-del_*tssL1*

To generate the *tssL1* deletion strain, 700-bp upstream and 700-bp downstream sequences of the *tssL1* gene were amplified by overlapping PCR with high-fidelity Q5 DNA polymerase (New England Biolabs) using primers A8-delL1-UP1bam (BamHI), A8-delL1-UP2, A8-delL1-DW3, and A8-delL1-DW4 (BamHI) (**Table S1**). The PCR product was cloned into pKNG101 suicide vector at the BamHI site, giving the mutator pKNG101-del_*tssL1*. pKNG101-del_*tssL1*, maintained in *E. coli* CC118 λpir, was further conjugated in *Achromobacter* spp. A8 using a protocol adapted from Sana et al. (2014). The mutant, in which the double-recombination events occurred and resulted in the nonpolar deletion of *tssL1* gene, was verified by PCR using external primers A8Del-check-F and A8Del-check-R (**Table S1**).

### Secretion Analysis

*Achromobacter* cultures grown at 28°C in LB for 24 h were back-diluted to an initial Optical Density at 600 nm (OD_600_) of 0.05 in LBor VB when required and subsequently grown to reach an OD_600_ of ~ 1.5. Cells were separated from culture supernatants by two consecutive centrifugations. A first centrifugation at 4,200 *g* for 10 min at 16°C from which the supernatant was submitted to a second one for 10 min at 9,000 *g* at 16°C. Secreted proteins were precipitated overnight with 15% trichloroacetic acid (w/v; TCA; Sigma Aldrich). Subsequently, the samples were spun at 13,000 g for 1 h at 4°C and washed with 90% cold acetone prior to be resuspended in Laemmli sample buffer (Bio-Rad). Whole cell lysates (WCL) were directly dissolved in Laemmli sample buffer after being spun at 4,200 *g* for 10 min.

### Sodium Dodecyl Sulfate–Polyacrylamide Gel Electrophoresis (SDS-PAGE), Immunoblotting, and Sera

Proteins samples were boiled, separated by homemade SDS-PAGE gels, and transferred to nitrocellulose membranes (Amersham Protan 0.2 µm, GE Healthcare) by Western blotting using Mini-protean and mini Trans-Blot system (Bio-Rad), respectively. The membranes were first saturated with PBST 5% milk and then incubated with the rabbit polyclonal antibodies directed against Hcp-1 or Ef-Tu (SIGMA). Finally, alkaline phosphatase–conjugated goat anti rabbit Immunoglobulin G (IgG) (Interchim) were detected with addition of 5-bromo-4-chloro-3-indolylphosphate (BCIP, Apollo Scientific Ltd) and nitroblue tetrazolium (NBT, Apollo Scientific Ltd) in alkaline buffer. When required, the FITC-conjugated polyclonal antibody Ef-Tu was analyzed with a LI-COR Odyssey imager.

### Dot Blot

Five microliters of sputum were spotted onto nitrocellulose membrane and air-dried. The membrane was immunoblotted as described above to assess the presence of Hcp in CF patient sputum. Signal intensity analysis was performed using ImageJ software. Dot signal intensities were determined using “plot lanes” commands corresponding to the area of each peak.

### Mass Spectrometry–Based Proteomics

The list of identified proteins provided in **Table 1** was established from mass spectrometry–based proteomics, on excised SDS-PAGE gel bands (duplicates) containing the purified proteins Hcp, as previously described (Santin et al., 2018). Slight modifications were brought: After in-gel trypsin digestion and LC-MSMS analysis on an Ultimate 3000 liquid chromatography coupled to a Q-Exactive plus mass spectrometer (ThermoFisher), spectra were processed by Proteome Discoverer software (ThermoFisher, version 2.4.1.15) using the Sequest HT algorithm with the databases extracted from Uniprot, *Achromobacter xylosoxidans* strain A8 (UP000006876 last modified July 24, 2020; 6,798 entries) and *Alcaligenes xylosoxidans xylosoxidans* (UP000040179 last modified March 23, 2021; 6138 entries). In this study, proteins were identified and filtered by a minimum number of Peptide Spectral Match of 2.

For quantitative proteomics, supernatants collected from the Ax4 CF strain grown in a rich medium (LB, the control condition) or in a CF-mimicking condition (CF-mimicking medium, the chemically defined Vogel-Bonner, VB) were collected in triplicate (2.5 ml with 4.2 to 5.9 OD_600_ units). These supernatants were TCA-precipitated and prepared for quantitative proteomic analysis by using the S-trap method according to the manufacturer’s procedure (ProtiFi). LC-MSMS injections (250 ng per sample) were performed and spectral data were processed, as previously described in Avilan et al. (2021), using the MaxQuant computational proteomics platform (version 1.6.5.0) for protein identification and quantification (Cox et al., 2011) and the Perseus statistical platform (version 1.6.5.0) (Tyranova et al., 2016) for statistical analysis. The same databases as described above were used for the search, supplemented with a set of 245 frequently observed contaminants. Quantifiable proteins (valid values) were defined as those detected in 50% in all conditions. Missing values were replaced using data imputation by randomly selecting from a normal distribution centered on the lower edge of the intensity values. To determine whether a given detected protein was specifically differential, a two-sample t-test was applied using a permutation-based False Discovery Rate (FDR)-controlled at 0.01 (250 permutations) and the results were illustrated in a volcano plot. The p-value was adjusted using a scaling factor s0 to a value of 1.

### Purification of TAX-1 Hcp

#### Cloning

PCRs were performed using Q5 high-fidelity DNA polymerase (New England Biolabs). Restriction enzymes and ligase were purchased from New England Biolabs and used according to the manufacturer’s instructions. Custom oligonucleotides were synthetized by Integrated DNA Technologies IDT. The *Achromobacter* spp. A8 chromosomal DNA was used as templates for PCRs. *E. coli* DH5α was used for cloning procedures. pCDF-Hcp1(HIS) was constructed by restriction cloning. Briefly, the hcp1 DNA fragment was amplified by PCR using oligonucleotides 05691-F (BamHI) and 05691-R (SacI) (**Table S1**). The PCR product was digested (BamHI and SacI), cleaned (Macherey-Nagel PCR cleaning kit), and cloned into the pCDF-Duet vector (NOVAGEN) by ligation using T4-DNA ligase. After 12 h of incubation at 16°C, the ligation mixture was transformed into competent *E. coli* DH5α, and recombinant strains were selected on the appropriate antibiotic (Sm). All constructs were verified by DNA sequencing.

#### Production and Purification

*E. coli* BL21(DE3) cells bearing pCDF-Hcp1(HIS) were grown at 37 °C in LB to an OD_600_ ~ 0.9 and gene expression was inducted with 0.5 mM IPTG for 16 h at 17°C. Cells were harvested; resuspended in Tris-HCl 50 mM (pH 8.0), 150 mM NaCl, and lysozyme (0.25 mg ml^−1^); and broken using an Emulsiflex (Avestin). Soluble proteins were separated from inclusion bodies and cell debris by centrifugation 30 min at 20,000*g*. The His-tagged fusions were purified using ion metal Ni2+ affinity chromatography (IMAC) using a 5-ml HisTrap column (Cytiva) and eluted with a step gradient of imidazole. Proteins were further separated on preparative Superdex 200 gel filtration column (Cytiva) equilibrated in 50 mM Tris-HCl (pH 8.0) and 150 mM NaCl. The fractions containing the purified protein were pooled and concentrated by ultrafiltration using the Amicon technology (Millipore, California, USA).

### Generation of Antibody

Synthetic polyclonal rabbit antibodies were designed by GeneScript for Hcp1 detection based on the following synthetic peptide epitope: RSATASTSGGHTAE. The anti-Hcp1 antibody has been tested by recombinant expression in *E. coli* of *Ax* Hcp1(**Figure S1A**).

### Bacterial Competition Assay

Rifampicin resistant MG1655 or PAO1 were used as prey in the competition assay. Attacker and prey cells were grown overnight in LB broth at 28°C, with rifampicin when required, washed twice with PBS and subsequently diluted in VB to an initial OD600 of 0.05, with rifampicin when required. After 24h incubation at 28°C in aerobic condition, cells were washed twice with PBS and normalized to an OD600 of 1. Attacker and prey cells were mixed in a 10:1 (attacker: prey) ratio and spun at 4,200 g for 5 min. To concentrate twice the mix, half of the supernatant was discarded prior to pelletresuspension. 10 μL competition drops as well as input strains alone were spotted onto dry VB agar (1.5% bactoagar). After 7 h incubation at 28°C, both the input and output of the competition wererecovered in PBS, normalized to an OD600 of 0.5 and serially diluted in PBS. 2.5 μL drops of thedilutions were spotted on LB agar plates containing rifampicin or PIA plates. Survival was observed after incubating plates 24 h at 37°C. Competition assays were done in triplicates. Representative picture is shown in **Figure 3** and other replicates in **Figure S6**. To determine the competitive index (ratio of MG1655-Rif to Achromobacter output divided by the ratio of MG1655-Rif to Achromobacter input), both species were counted before and after the coincubation. The mixed species were serially diluted and plated onto LBagar plates containingrifampicin or PIA to enumerate prey and attacker bacteria. The colony forming units were counted after 24h or 48h (for Achromobacter species) incubation at 37°C.

### *A. xylosoxidans* Adhesion and Internalization Assay

Human adenocarcinomic alveolar epithelial cells A549 (ATCC^®^ CCL-185) were cultured in Iscove’s Modified Dulbecco’s Medium (IMDM; Thermo Scientific) supplemented with 10% (v/v) heat-inactivated fetal bovine serum (FBS) and 1% penicillin-streptomycin at 37°C in humidified air containing 5% CO_2_. Cells were plated at 2.10^5^ cells per well in 24-well culture plates the day before infection. To quantify the intracellular invasion of the different *P. aeruginosa* strains, we used the gentamicin survival assay. Infections with the different strains were performed in IMDM 10% FBS without antibiotic during 4, 8, and 48 h at a multiplicity of infection (MOI) of 1:1. *A. xylosoxidans* was cultivated at 28°C in LB for 24 h, washed in phosphate buffered saline (PBS), diluted to an initial OD_600_ of 0.005, and then cultured in VB for 24 h. Bacterial strains were diluted in IMDM 10% FBS to obtain the desired MOI and start the infection. After 4, 8, or 48 h of infection, the supernatant of the wells was removed and cells were washed with PBS.

For the adhesion assay, the cells were washed four times with PBS to remove non-adherent bacteria from the supernatant before lysis using 1% Triton X-100 (Thermo Scientific). Adherent bacteria were counted after serial dilution of cell lysate in PBS and LB agar plate isolation.

For the internalization assay, the infections were stopped using gentamycin (100 µg/ml) diluted in IMDM 10% FBS for 24 h. Cells were washed three times with PBS before lysis using 1% Triton X-100 (Thermo Scientific). As for the adhesion assay, internalized bacteria were counted after serial dilution of cell lysate in PBS and LB agar plate isolation. All assays were performed in quadruplicate. Raw data were transformed into percentage of the internalized bacteria per well over the mean count of adhered bacteria according to the bacterial subtype (A8 wild-type or A8Δ*tssL*) and the duration of the infection (4, 8, and 48 h).


$$\frac{internalized\;bacteria\;\left(CFU/mL\right)}{mean\;of\;adherent\;bacteria\;\left(CFU/mL\right)}\;\times 100$$

To monitor the cell monolayer integrity and the virulence of each strains, lactate deshydrogenase leakage into the supernatant was quantified using the cytotoxicity detection kit (Roche) according to the manufacturer’s instruction. The percentage of cell mortality was determined by counting dead cells over live cells per well using trypan blue after trypsinization.

### Time-Kill Assay

Bacterial strains were diluted in Mueller Hinton (MH) and VB broth at 10^6^ CFU/ml and placed in an incubator shaker at 37°C with gentamicin (100 µg/ml). Living bacteria in the medium were counted after serial dilution in PBS at specific times: prior antibiotic exposition and after 1, 3, and 8 h of antibiotic exposition.

### Statistical Analysis

All statistical analysis and graphical representations were performed using Prism 9 software (Graph-Pad Software, San Diego CA).

## Data Availability Statement

The original contributions presented in the study are included in the article/**Supplementary Material**. Further inquiries can be directed to the corresponding authors.

## Author Contributions

ED and FG conceived the project. ED, FG, GI, MLG, MV, RL, PM, AD, TG, PT, PG, and RD designed the experiments. ED and FG wrote the manuscript with contribution from all the authors. All authors contributed to the article and approved the submitted version.

## Funding

This work was funded by the Centre National de la Recherche Scientifique, the Aix-Marseille Université, and grants from the Agence Nationale de la Recherche (ANR-18-CE11-0023-01), from Vaincre La Mucoviscidose and Association Gregor Lemarchal (RC20200502657) to FG and ED, and from the “Association Limousine d’Aide aux Insuffisants Respiratoires” (ALAIR) to FG. Marseille Protéomique (MaP) is supported by IBiSA (Infrastructures en Biologie Santé et Agronomie) and Aix Marseille Université. ED is supported by the Institut National de la Santé et de la Recherche Médicale (INSERM).

## Conflict of Interest

The authors declare that the research was conducted in the absence of any commercial or financial relationships that could be construed as a potential conflict of interest.

## Publisher’s Note

All claims expressed in this article are solely those of the authors and do not necessarily represent those of their affiliated organizations, or those of the publisher, the editors and the reviewers. Any product that may be evaluated in this article, or claim that may be made by its manufacturer, is not guaranteed or endorsed by the publisher.

## Supplementary Material

Supplementary Figure 1 Specificity of the antibodies targeting the TAX-1 Hcp. **(A)** Immunoblot analysis on whole-cell lysates (WCL) from *E. coli* BL21 expressing Hcp-1 (T+) or not (T−), and supernatant samples (SN) from Ax4 grown in LB or VB, as well as purified Hcp-1^His^ protein. This analysis confirms the specificity of the Hcp-1 antiserum used in this study. **(B)** Immunoblot analysis of culture supernatants and whole-cell lysates from A8, Ax4, and Ax6 strains grown in LB or VB medium. The equivalent of 0.1 and 2 OD units were loaded for WCL and supernatant fractions, respectively, and analyzed with Hcp-1 antiserum. The position of Hcp-1 is indicated on the right. Molecular weight markers (in kilodaltons) are indicated on the left. **(C)** Immunoblot analysis with Hcp-1 antiserum of culture supernatants from A8, Ax4, and PAO1 strains grown in VB.

Supplementary Figure 2 Growth curves of A8 and A8Δ*tssL.* Three independent cultures of A8 strain (black) and A8Δ*tssL* (gray) were grown in LB (solid line) or VB (dashed line) medium. Lines and filled area represent the averages and standard deviation, respectively.

Supplementary Figure 3 Sequence alignment of AXYL_05707 with predicted homologs Tle5 and Tle5b from *P. aeruginosa*. The multiple sequence alignment was generated with the web server T-Coffee using PSI-Coffee mode. The conserved catalytic residues of HKD domains (box) are indicated above the alignment with black arrows.

Supplementary Figure 4 Growth of input strains as controls for. **(A)** Picture of a representative growth of strains used in competition assay on PIA or LB-Rif agar plates. 10-fold serial dilution drops are spotted after incubation on VB agar during 6 h at 28°C. This analysis shows no growth difference between A8 and A8Δ*tssL*. **(B)** LDH released by A549 after 4, 8, and 48 h of infection at multiplicity of infection (MOI) 1:1. **(C)** Percentage of A549 cells mortality counted with trypan blue after 4, 8, and 48 h of infection at MOI 1:1.

Supplementary Figure 5 Volcano Plot of differentially secreted proteins of Ax4 grown in LB versus VB. TAX-1 proteins are shown in red, proteins from T3SS in green and those related to flagella in blue.

Supplementary Figure 6 Deletion of *tssL* affects A8 competition against *E. coli* and PAO1. Picture of replicates of competition assays showing survival of MG1655-Rif **(A)** or PAO1-Rif **(B)** after co-incubation with *Achromobacter* A8 or A8Δ*tssL*. Prey and attacker were mixed in a 1:10 ratio and incubated 6 h at 28°C on VB agar. Surviving preys are observed after spotting 10-fold serial dilutions of the mix on LB agar containing rifampicin.

Supplementary Figure 7 T6SS expression in clinical isolates. qRT-PCR analysis of *tssM* **(A)** and *hcp* **(B)** expression in clinical isolates from CF sputum (x5), blood infection (x2), and environmental (x2) origins. Data from three biological replicates are normalized with the housekeeping gene *nrdA*.

Supplementary Table 1 Primers used in the study.

Supplementary Table 2 Hcp detection by spectrometry-based proteomics in A8, Ax4, and Ax6 strains.
