# Supplementary figures and images for: Characterization of the Achromobacter xylosoxidans Type VI Secretion System and Its Implication in Cystic Fibrosis
Source: Front Cell Infect Microbiol. 2022 Jun 16;12:859181. doi: 10.3389/fcimb.2022.859181 (PMC9245596; doi:10.3389/fcimb.2022.859181)

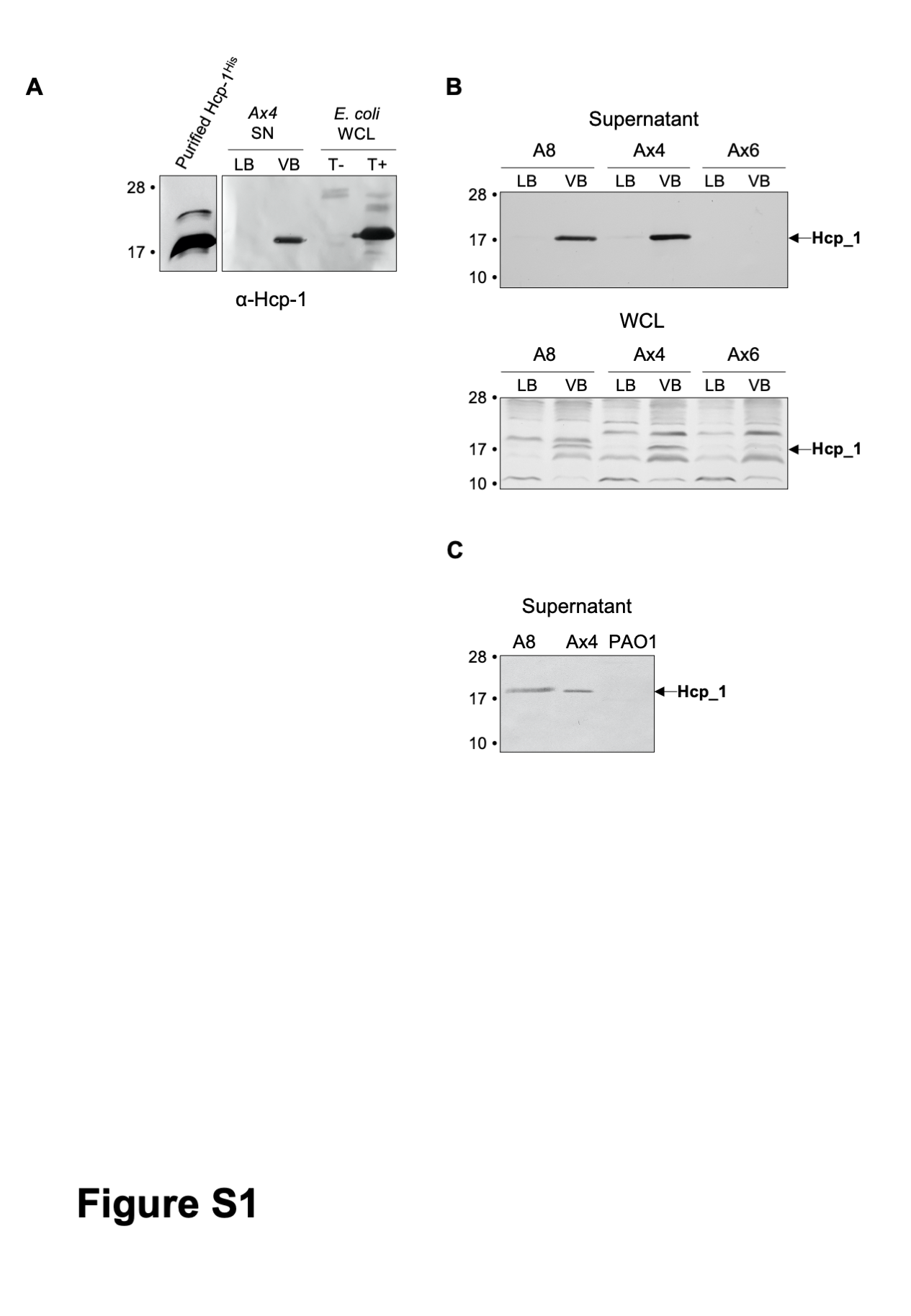

Supplement: Supplementary Figure 1 — Specificity of the antibodies targeting the TAX-1 Hcp. (A) Immunoblot analysis on whole-cell lysates (WCL) from E. coli BL21 expressing Hcp-1 (T+) or not (T−), and supernatant samples (SN) from Ax4 grown in LB or VB, as well as purified Hcp-1His protein. This analysis confirms the specificity of the Hcp-1 antiserum used in this study. (B) Immunoblot analysis of culture supernatants and whole-cell lysates from A8, Ax4, and Ax6 strains grown in LB or VB medium. The equivalent of 0.1 and 2 OD units were loaded for WCL and supernatant fractions, respectively, and analyzed with Hcp-1 antiserum. The position of Hcp-1 is indicated on the right. Molecular weight markers (in kilodaltons) are indicated on the left. (C) Immunoblot analysis with Hcp-1 antiserum of culture supernatants from A8, Ax4, and PAO1 strains grown in VB. [file Image_1.tiff]

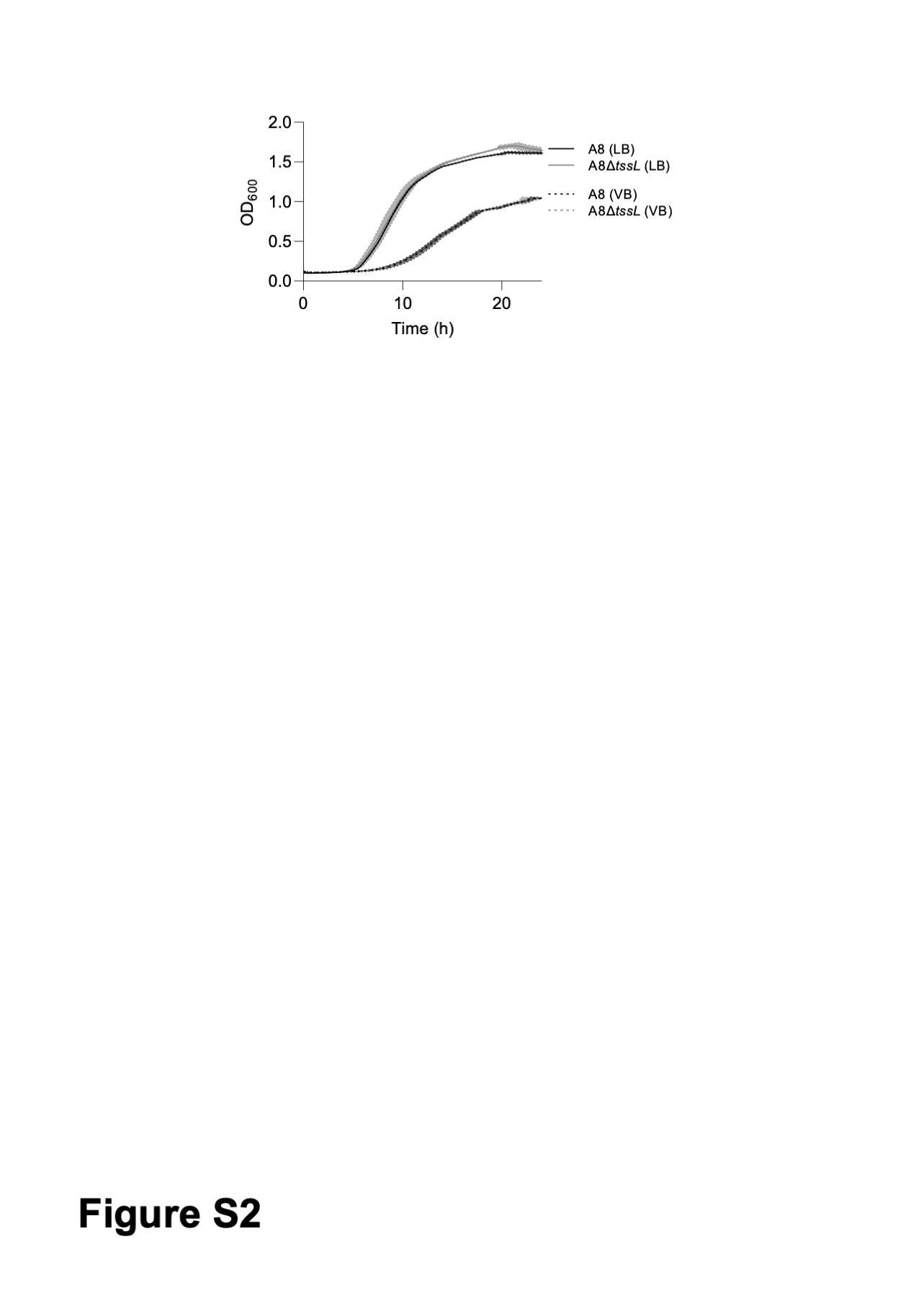

Supplement: Supplementary Figure 2 — Growth curves of A8 and A8ΔtssL. Three independent cultures of A8 strain (black) and A8ΔtssL (gray) were grown in LB (solid line) or VB (dashed line) medium. Lines and filled area represent the averages and standard deviation, respectively. [file Image_2.tiff]

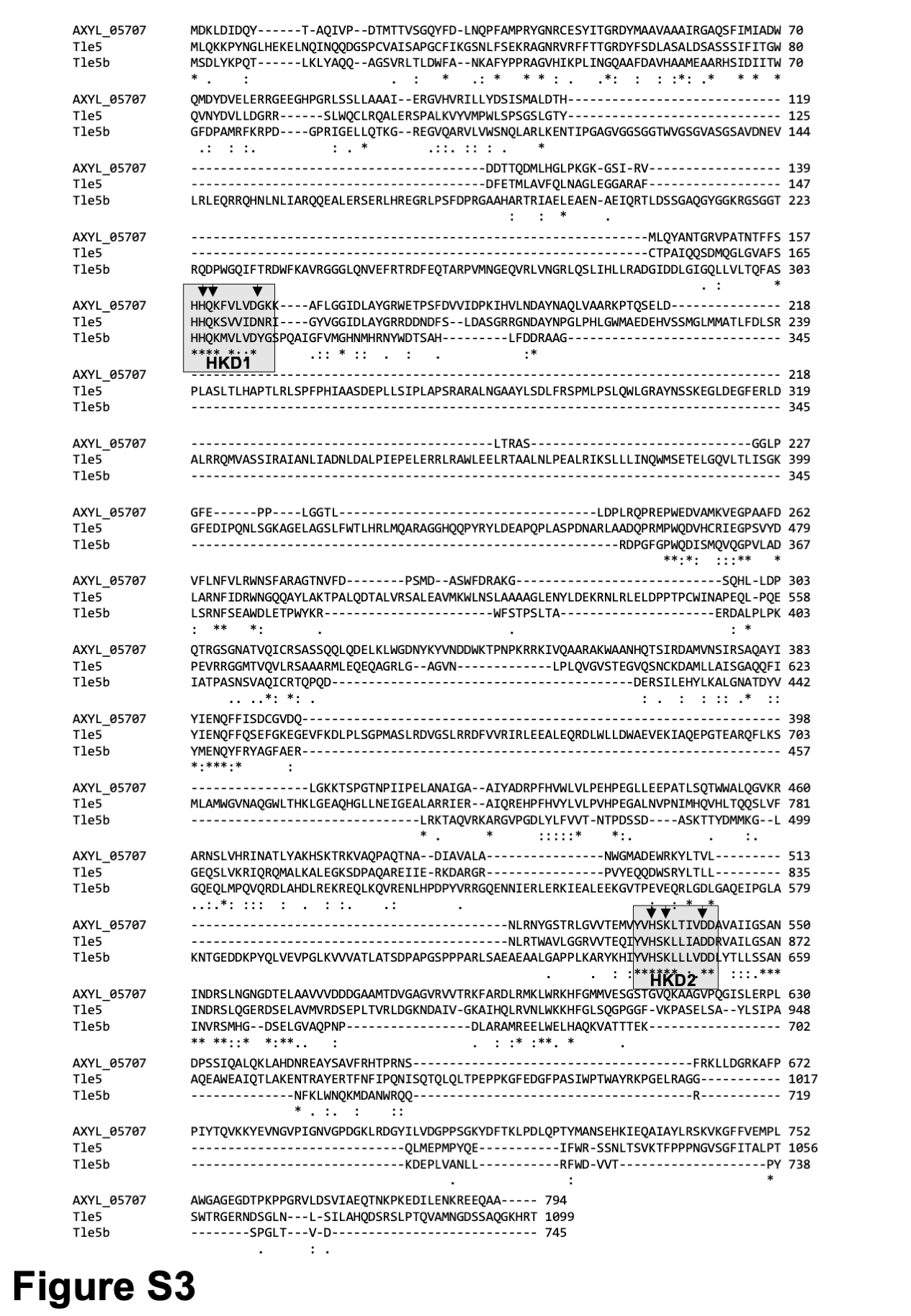

Supplement: Supplementary Figure 3 — Sequence alignment of AXYL_05707 with predicted homologs Tle5 and Tle5b from P. aeruginosa. The multiple sequence alignment was generated with the web server T-Coffee using PSI-Coffee mode. The conserved catalytic residues of HKD domains (box) are indicated above the alignment with black arrows. [file Image_3.tiff]

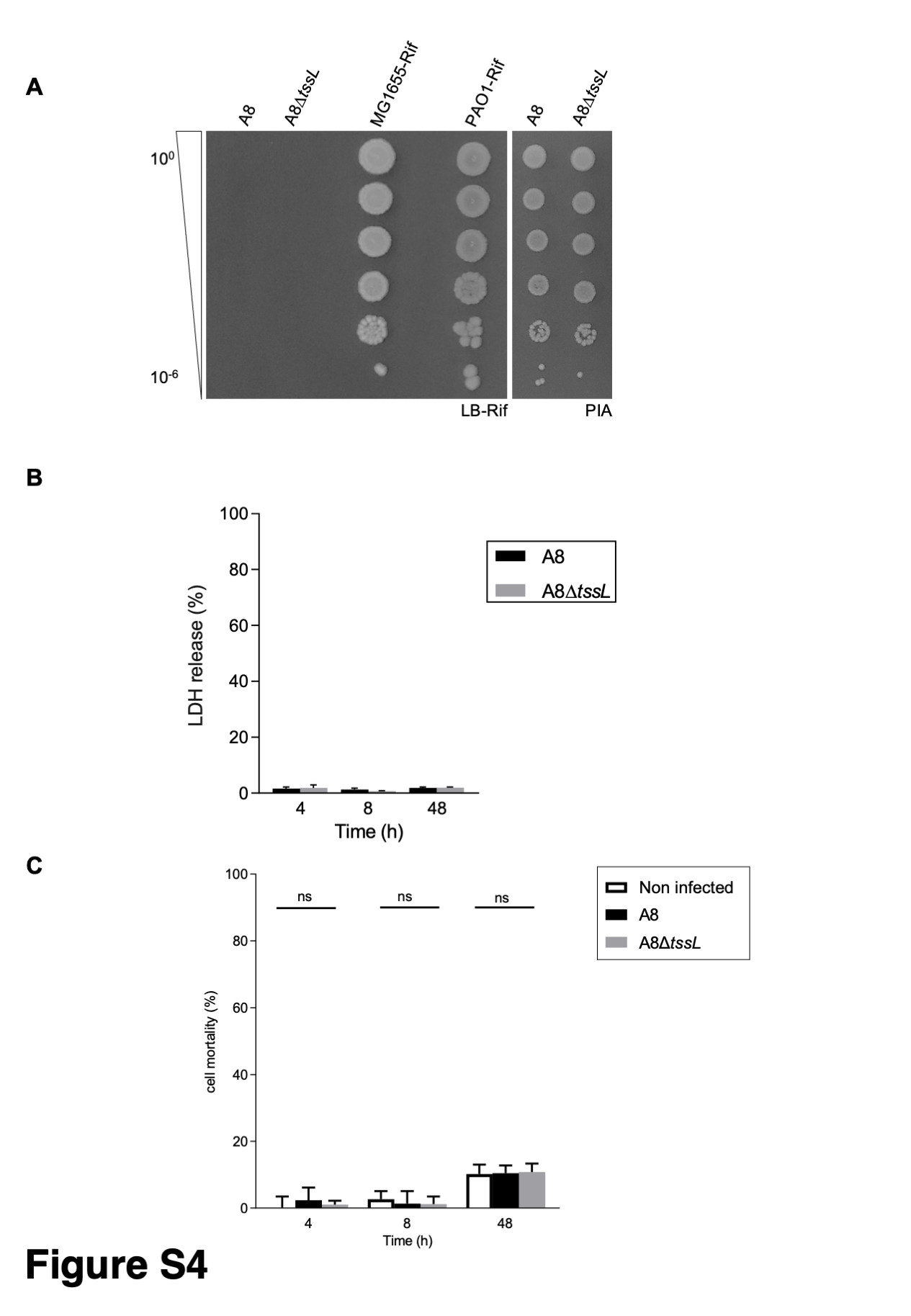

Supplement: Supplementary Figure 4 — Growth of input strains as controls for. (A) Picture of a representative growth of strains used in competition assay on PIA or LB-Rif agar plates. 10-fold serial dilution drops are spotted after incubation on VB agar during 6 h at 28°C. This analysis shows no growth difference between A8 and A8ΔtssL. (B) LDH released by A549 after 4, 8, and 48 h of infection at multiplicity of infection (MOI) 1:1. (C) Percentage of A549 cells mortality counted with trypan blue after 4, 8, and 48 h of infection at MOI 1:1. [file Image_4.tiff]

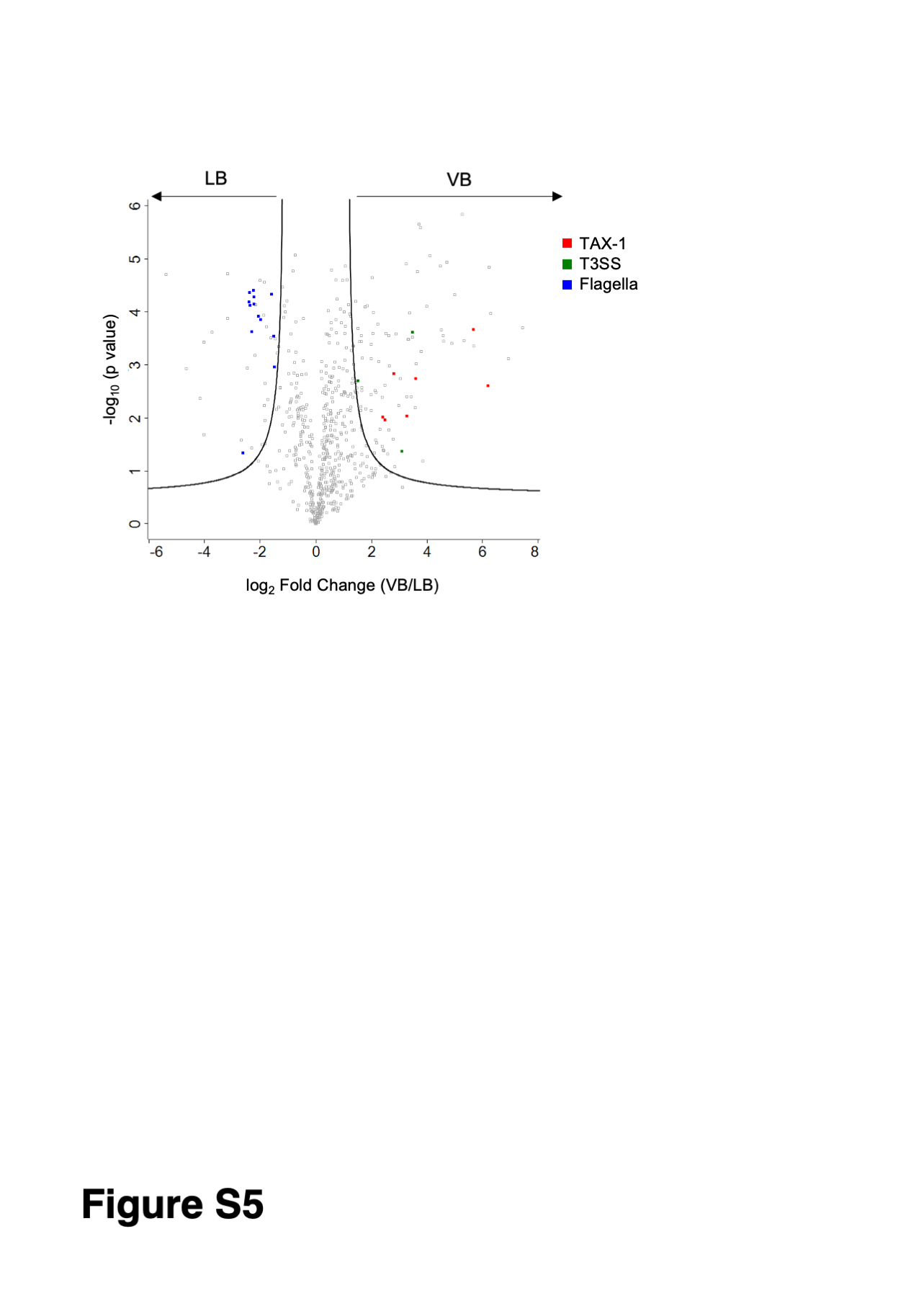

Supplement: Supplementary Figure 5 — Volcano Plot of differentially secreted proteins of Ax4 grown in LB versus VB. TAX-1 proteins are shown in red, proteins from T3SS in green and those related to flagella in blue. [file Image_5.tiff]

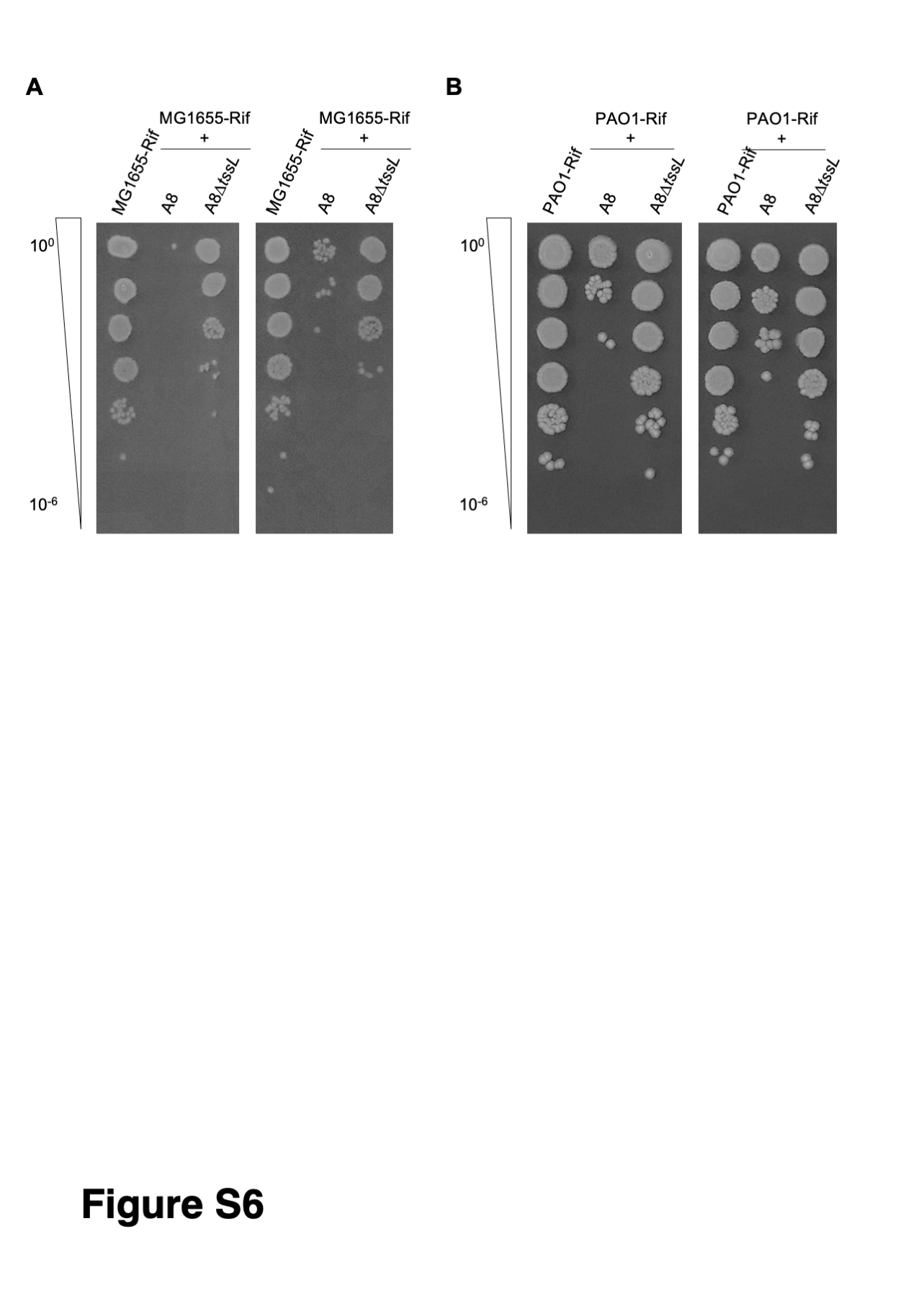

Supplement: Supplementary Figure 6 — Deletion of tssL affects A8 competition against E. coli and PAO1. Picture of replicates of competition assays showing survival of MG1655-Rif (A) or PAO1-Rif (B) after co-incubation with Achromobacter A8 or A8ΔtssL. Prey and attacker were mixed in a 1:10 ratio and incubated 6 h at 28°C on VB agar. Surviving preys are observed after spotting 10-fold serial dilutions of the mix on LB agar containing rifampicin. [file Image_6.tiff]

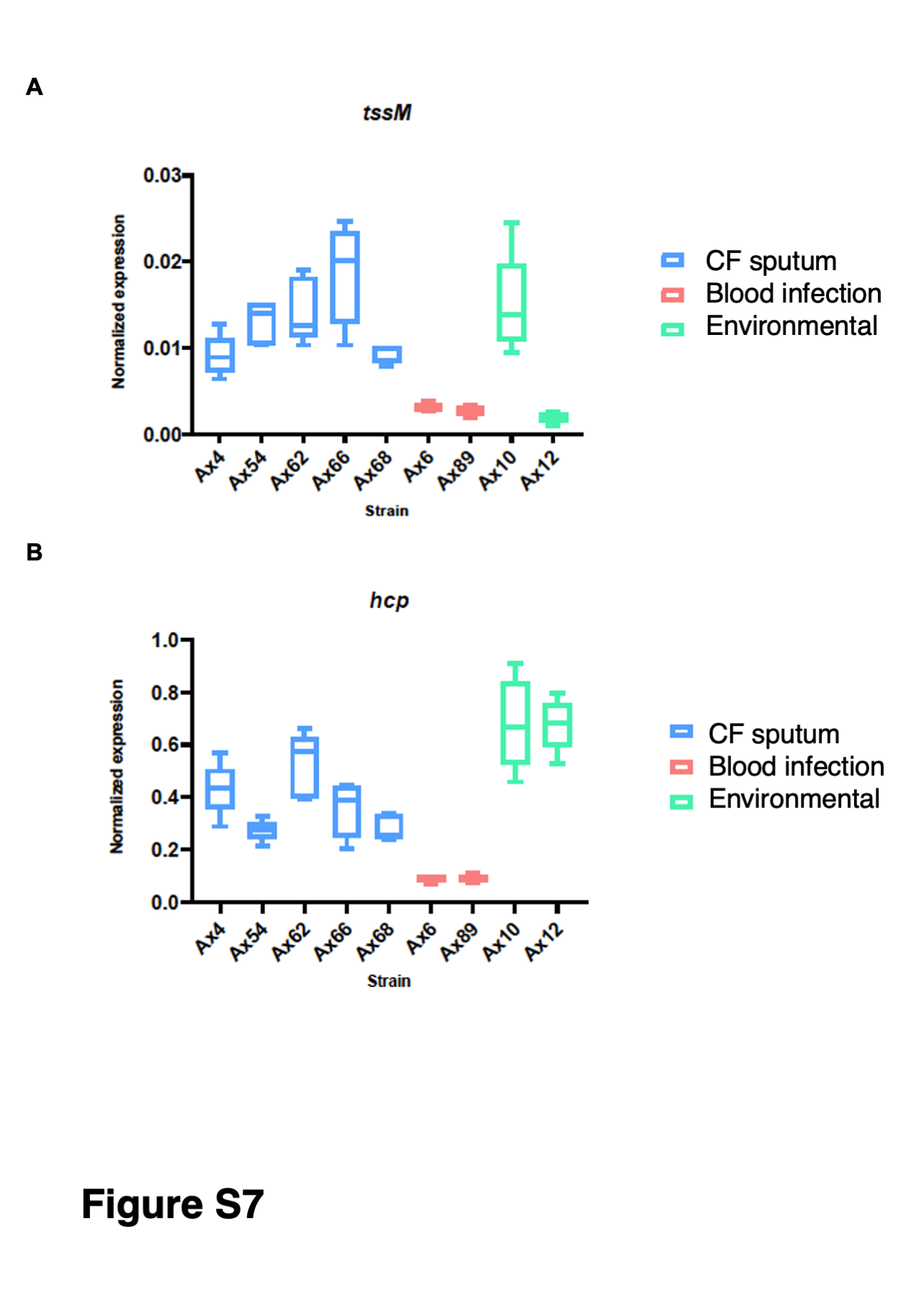

Supplement: Supplementary Figure 7 — T6SS expression in clinical isolates. qRT-PCR analysis of tssM (A) and hcp (B) expression in clinical isolates from CF sputum (x5), blood infection (x2), and environmental (x2) origins. Data from three biological replicates are normalized with the housekeeping gene nrdA. [file Image_7.tiff]

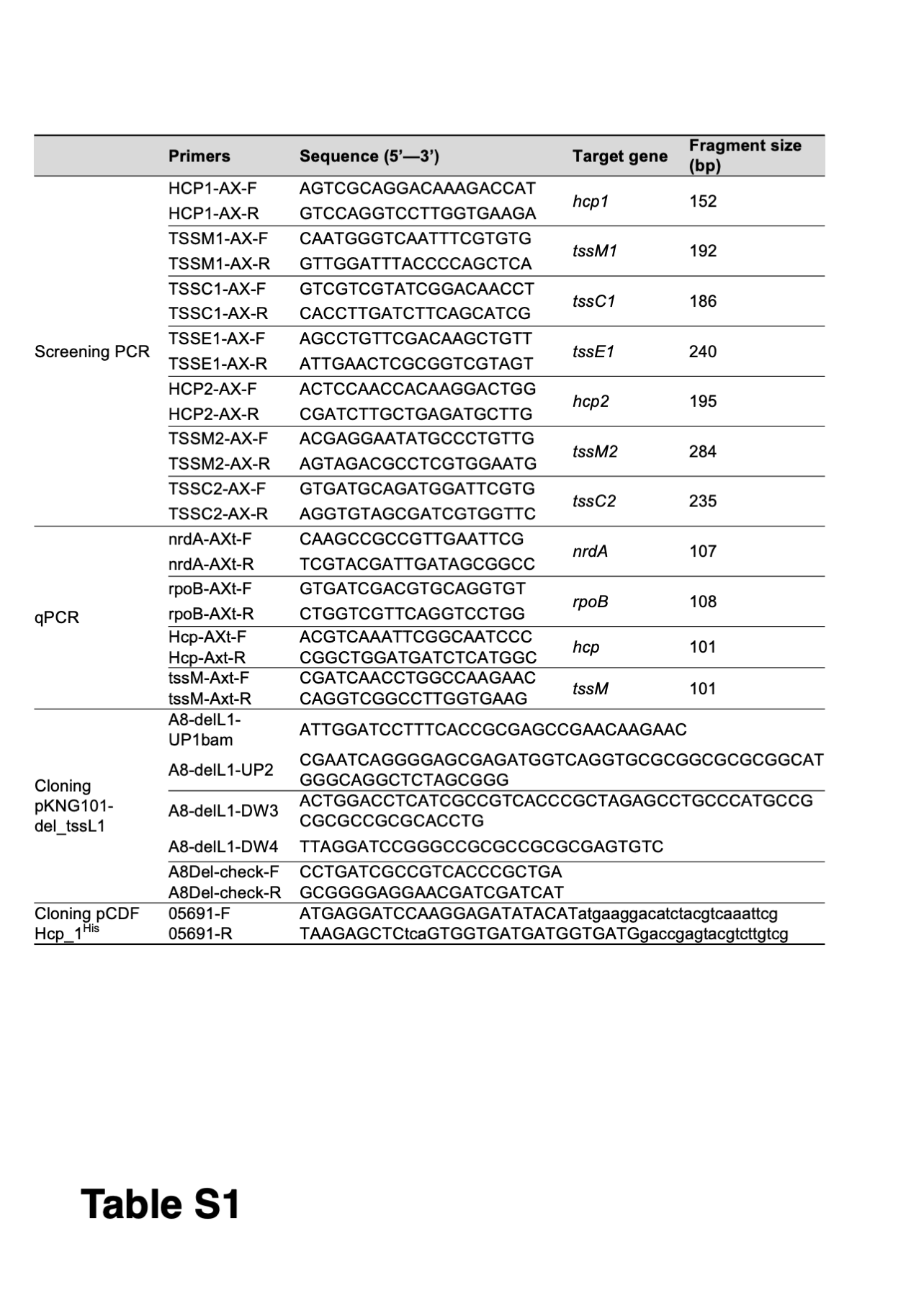

Supplement: Supplementary Table 1 — Primers used in the study. [file Image_8.tiff]

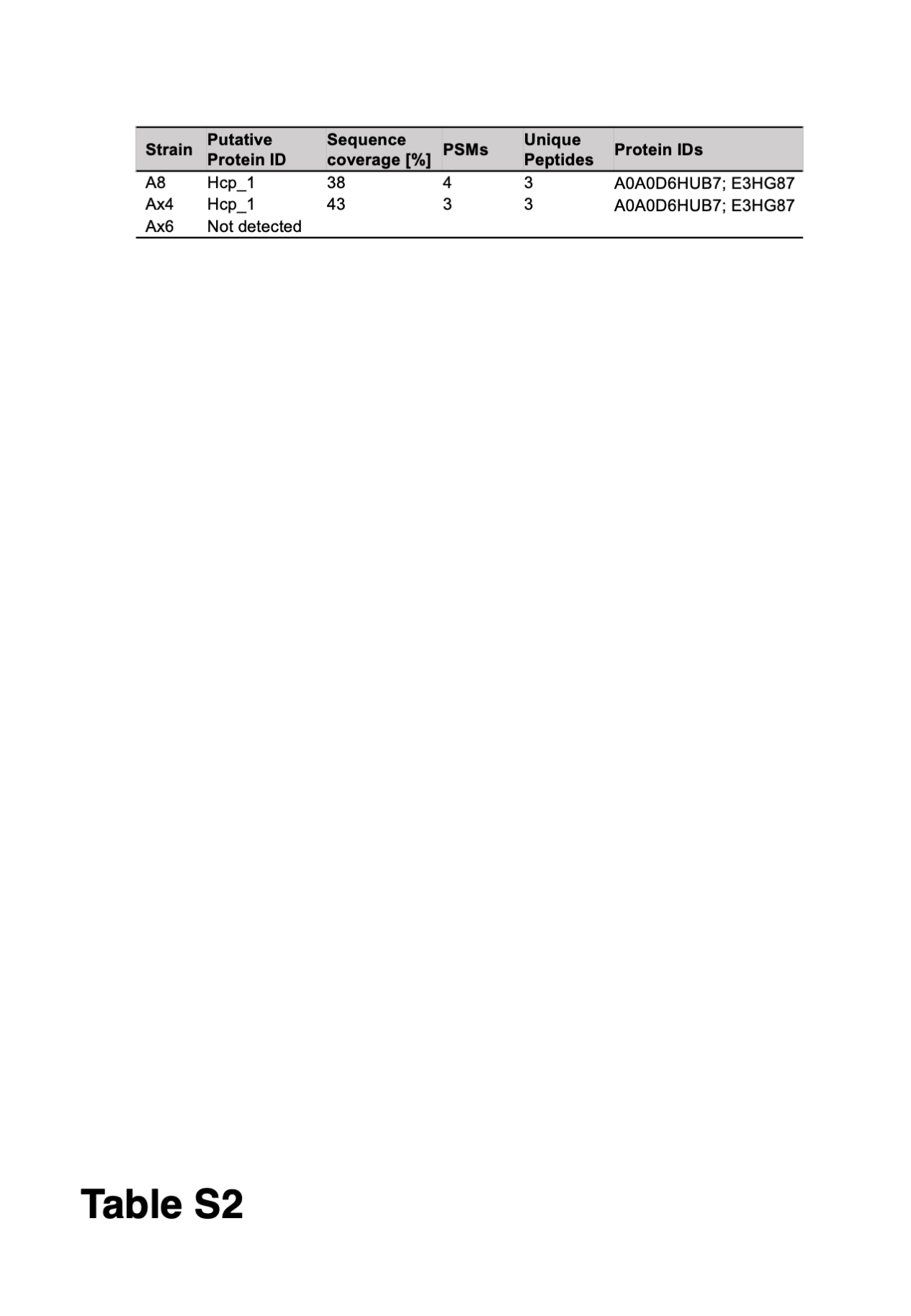

Supplement: Supplementary Table 2 — Hcp detection by spectrometry-based proteomics in A8, Ax4, and Ax6 strains. [file Image_9.tiff]
